# Holobricks: modular coarse integral holographic displays

**DOI:** 10.1038/s41377-022-00742-7

**Published:** 2022-03-16

**Authors:** Jin Li, Quinn Smithwick, Daping Chu

**Affiliations:** 1 Centre for Photonic Devices and Sensors, University of Cambridge, 9 JJ Thomson Avenue, Cambridge, CB3 0FA UK; 2 Disney Research, 521 Circle 7, Glendale, CA 91201 USA

**Keywords:** Displays, Integrated optics

## Abstract

Here, we propose and demonstrate a modular holographic display system that allows seamless spatial tiling of multiple coarse integral holographic (CIH) displays called “holobricks”. A holobrick is a self-contained CIH module enclosing a spatial light modulator (SLM), a scanner, and periscopic coarse integral optics. Modular CIH uses a coarse pitch and small area but high-bandwidth SLM in conjunction with periscopic coarse integral optics to form the angularly tiled 3D holograms with large viewing areas and fields of view. The creation of periscopic coarse integral optics prevents the optical system from being larger than the holographic image and allows the holographic fringe pattern to fill the entire face of the holobrick. Thus, multiple holobricks can be seamlessly abutted to form a scalable spatially tiled holographic image display capable of both wide field-of-view angle and arbitrary large-size area. We demonstrate an initial prototype that seamlessly tiles two holobricks each with 1024 × 768 pixels, 40° FOV, full color, 24 fps, displaying 2D, 3D holographic stereograms, and full parallax 3D CGI Fresnel holograms.

## Introduction

Holographic displays are considered the ultimate display technology because they can generate arbitrary wavefronts to provide imagery with all-essential three-dimensional (3D) realistic visual cues of a scene^1–9^, and therefore have great future market potential^10–13^. To generate a simultaneous large-size and wide-viewing-angle holographic display, a spatial light modulator (SLM) must present a holographic fringe pattern of sufficient space-bandwidth product (SBWP) – the product of fringe pitch and modulation area. Such a fringe pattern is thereby capable of generating a holographic image with a gigantic optical invariant etendue or optical extent, the product of field-of-view (FOV) angle and image area. Unfortunately, the information amount of a holographic image with an enormous optical invariant etendue is significantly higher than the modulation capacity of present SLMs with their coarse pixel pitches and small display areas, i.e. low SBWP.

A Coarse Integral Holography (CIH) framework has been presented to overcome the low SBWP of current SLMs for use in holographic displays^14–16^. CIH utilizes coarse integral optics to allow us angularly tile many scanned holograms generated by a coarse pitch, small area but high frame rate (i.e. low SBWP, but high bandwidth) SLM, such as a digital-micro-mirror device (DMD)-based SLM to create a large format wide-viewing-angle full-frame rate (24 fps) holographic display (i.e., large optical extent at modest frame rates). Further development concentrated on making the CIH display *scalable*, further increasing the information content of the hologram and improving the display’s capability to distribute the hologram’s light and information. This was accomplished by modifying the architecture to incorporate multiple SLMs and multiple scanners with larger mirrors and greater scan speed. However, the optics’ information-handling capabilities, as measured by its effective f/# (the quotient of the focal length and the effective aperture) then becomes the limiting factor that governs the largest size and FOV of reconstructed holographic images.

To transcend the current size and corresponding information content of the holographic 3D image, we need to make the CIH architecture *modular*, so that multiple displays can be appended together to increase the information content of the final holographic image. To do so, we modify the scanned coarse integral optics by incorporating a periscopic relay array so multiple CIH displays can be seamlessly spatially tiled, thereby creating so called “holobricks.” Each holobrick is a self-contained holographic display module with SLMs, scanners, and periscopic coarse integral optics, each creating a wide-field modestly sized holographic image equal to the size of the display module. Multiple spatially tiled holobricks can be seamlessly abutted and modularly arranged to create a contiguous large extent wide FOV final holographic video image.

### Theory

A measure of the information content of the holographic fringe pattern and the spatial light modulator is SBWP. Similarly, a measure of the information content of a holographic 3D image is the optical extent (i.e., optical invariant etendue), *AΩ*. A single holographic fringe pattern and an SLM can both be characterized by their space bandwidth products. The space bandwidth product can be expressed by

1 $${\mathrm{SBWP}} = \left( {4 \times S_x \times S_y} \right)/\left( {d_x \times d_y} \right)$$

where *S*_*x*_ is the horizontal size (width) of the holographic pattern, *S*_*y*_ is the vertical size (height) of the holographic pattern, *d*_*x*_ is the fringe period span of the holographic pattern in the horizontal dimensional direction, and *d*_*y*_ is the fringe period span of the holographic pattern in the vertical dimensional direction.

Similarly, the diffracted light forming the holographic image created by illuminating the SLM displaying the holographic pattern can also be represented with regard to its information content. The optical extent^17^ of the holographic image, denoted by *AΩ*, is defined by

2 $$\begin{array}{l}A\Omega = S_x \times S_y \times \cos \left( {0.5\left( {\varphi _{1x} + \varphi _{2x}} \right)} \right) \\ \quad\quad\;\;\;\times \left( {\varphi _{1x} - \varphi _{2x}} \right) \times \left( {\cos \theta _{1y} - \cos \theta _{2y}} \right)\end{array}$$

where *φ*_1*x*_ and *φ*_2*x*_ denote the horizontal boundary values of diffracted rectangular FOV solid angle. Accordingly, *θ*_1*y*_ and *θ*_2*y*_ represent the vertical boundary values of diffracted rectangular FOV solid angle.

Because information is conserved, the space-bandwidth product of the holographic fringe pattern and SLM, and the optical extent of the holographic image and its diffracted light traveling through the optical system are related. The diffraction equation relates the pitch/period of the holographic pattern/SLM pixels to the diffracted field of view of the hologram. The diffraction viewing zone of an SLM is determined by

3 $$\Phi = \sin ^{ - 1}\left( {\lambda _{{\mathrm{laser}}}/2p_{x/y}} \right)$$

where *λ*_laser_ is the reconstructed laser beam wavelength utilized in the holographic display system, *p*_*x/y*_ represents the pixel size (horizontal or vertical direction) of the SLM.

For holographic video, a stream of holographic patterns must be presented on the SLM to create sequences of holographic images at a sufficient rate (e.g. 15 fps) to provide the impression of consistent smooth motion. The rate of information, or bandwidth, is equal to the information per second. Since the information content of the holographic pattern or SLM image is described by the SBWP, the bandwidth (BW) of the holographic video or SLM’s pattern rate can be described by its SBWP per second, or SBWP × frame/pattern rate.

4 $${\mathrm{BW}} = {\mathrm{SBWP}} \times F_r = \left( {4 \times S_x \times S_y} \right)/\left( {d_x \times d_y} \right) \times F_r$$

where BW is the bandwidth, SBWP is the space-bandwidth-product. *S*_*x*_, *S*_*y*_, *d*_*x*_, and *d*_*y*_ are the same representation as Eq. (1), and *F*_*r*_ denotes the frame rate (fps).

Similarly, any optical elements (mirrors, lenses) or active opto-mechanics (scanners) must also be capable of accommodating, manipulating, and transmitting the information contained in the hologram’s caustic as it travels through the optical system, and at acceptable frame rates for holographic video. Therefore, the appreciation of the information content and rates of the desired holograms and the information capabilities of optical components of the holographic video display are important in designing systems capable of displaying large area, wide FOV, video-rate holograms.

For example, the horizontal or vertical pixel pitches of the SLMs, e.g. DMDs^18^ and LCOS-SLMs^19^, are only 5–13 μm. From Eq. (3), a single SLM with a square pixel size of 12 μm × 12 μm can produce a diffracted viewing zone of 1.31 × 1.31 when the wavelength of the illumination beam of the holographic display is 550 nm. From Eq. (1) and Eq. (3), the single SLM with 1024 × 1024 pixels has an SBWP of 4.19 × 10^6^ and its optical invariant etendue is 259.12 mm^2^ deg^2^.

For comparison, a moderately sized hologram of 60 mm × 60 mm is displayed with the FOV of 30° × 6°, where the desired holographic image holds the required optical extent of ~10^6^ mm^2^ deg^2^. The targeted optical extent attains proximately 3859 times higher than the actual SLM’s display capabilities. Therefore, the information content of the desired hologram with a tremendous optical extent obviously transcends the SLM display capabilities due to the SLM’s low SBPW. Accordingly, the small diffraction angle of the SLM with the limited number of pixels fundamentally derives into displaying a realistic small-size and narrow-FOV 3D holographic scene.

### Related technologies

To accept the vast optical invariant produced by desirable holograms, many 3D holographic display systems have been presented to solve the aforementioned technical issues of current SLMs^20–22^. Lum et al^21^. constructed a holographic display system based on optical scan-tiling of high-speed SLMs to increase the pixel counts to implement a large-size hologram display, but with a small FOV angle. Smalley et al^23^. employed an anisotropic leaky-mode modulator and a horizontal polygon scanner to achieve holographic displays with a large FOV. Joonku et al. developed a dynamic holographic display approach enabling a wide-FOV angle by incorporating multiple SLMs that are assembled using a curved array structure^24^. Jia et al^25^. exploited rotational tiled gratings in conjunction with a vertical diffuser to implement a holographic display capable of yielding a wide-viewing-angle zone in both horizontal and vertical directions. A*STAR group developed a scanned spatial tiling of 24 recovered sub-holograms to create the large-size holographic display^26^. Takaki et al^27–32^. investigated different horizontal scanning holographic displays to obtain a wide-viewing-zone angle. Yaraş et al^33^. tiled multiple SLMs in a circular configuration to construct a circular holographic video display system to enhance the viewing angle, but with limited scalability of image size. Sasaki et al^34^. demonstrated a holographic display system using multiple SLMs to achieve a large-size image size, but narrow viewing angle displays. Although the aforementioned methods can provide enhanced performance over that of a single SLM, they are not tileable and scalable, making it difficult in the simultaneous implementation of wide viewing angle and large size.

MIT Mark II created a scalable holographic display^35^. Its traveling holographic fringes were created using two 18-channel Acousto-Optical Modulators (AOMs) on a single large tellurium dioxide crystal. The traveling holographic fringes were descanned and the aperture enlarged using a bank of six (6) ganged horizontal scanners at the Fourier plane. The display was scalable; to increase the information content (number of holographic lines and the field of view), they could increase the number of bulk AOMs to increase the number of horizontal lines and/or scanners to expand the FOV. However, the system is limited by f/# of the optics to be able to accept and demagnify the hologram. Although holographic scan lines are tiled, multiple displays could not be tiled as the final lens is larger than the generated holographic image.

Qinetic used a combination of electronically addressed SLM (EASLM), an optically addressed SLM (OASLM) in combination with replication optics to create tileable holographic display systems^36^. The holographic pattern presented on the EASLM is optically duplicated using shuttered replication optics (a large collimating lens and an imaging lenslet array with corresponding array of optical shutters) onto the OASLM. Sub-sections of the desired holographic patterns are presented on the EASLM with corresponding shutters opened, allowing a complete montage of holographic fringe patterns to be written onto the OASLM. With the large fringe pattern written, a reconstruction beam illuminates the OASLM to create a holographic image. The OASLM is the size of the holobrick, so they can be tiled to form even larger holographic patterns. The combination of EASLM, OASLM, and shutters provided a valuable reference for tileable holographic display systems.

## Results

### Proposed coarse integral holography architecture

A Coarse Integral Holography (CIH)^14–16^ has been previously demonstrated by our group to achieve a full parallax, full-color holographic display endowed with a large SBWP (moderate area, wide horizontal FOV) at video frame rates. The CIH displays form wide viewing angle holographic imagery (high optical invariant etendue) by utilizing Coarse Integral Optics (CIO) to implement angular tiling of the fields-of-view of multiple low optical extent holograms generated by scanning a small SBWP but large BW (e.g. high kHz pattern rates) SLM. A solid-state CIH, a dynamic CIH (dCIH), and scalable full-bandwidth dCIH approaches are developed to improve the SBWP of holographic displays, details of which can found in Supplementary S1.

#### Holobricks: seamlessly modular CIH displays

However, an issue in the current full-bandwidth dCIH system still exists that limits the scaling of the CIH displays to ultra-large sizes and wide FOVs in both directions. We can add more SLMs to increase the information content of the hologram patterns, and additional scanners to handle and distribute that information at the appropriate rates. Ultimately, however, the lenses in the coarse integral optics then become the limiting factor. Specifically, the f-number (f/#) of the common transform lens must be small to handle the large area and wide fields-of-view of holograms with large optical extents.

5 $$f/\# = f_{lens}/D_{lens}$$

where *f/#* is the f-number of the lens of the coarse integral optics*, f*_lens_ is the focal length (FL) of the lens of the coarse integral optics, and *D*_lens_ is the effective aperture diameter of the lens of the coarse integral optics. The f-number is related to the number of resolvable points a lens can produce, and hence, its information handling capability.

After reaching the information and bandwidth limits of the SLM, scanners, and optics, in order to further promote the size and/or viewing angle (i.e., information) of the hologram, combining multiple optimized systems is needed. However, the current sCIH and dCIH displays are not modular or spatially tileable without seams.

For a holographic display system to be modular and capable of forming a seamless montage of holographic patterns and images, its mechanism cannot be bigger than the pattern or image itself. In the current sCIH and dCIH systems, the large output transform lens is much larger than the final angularly tiled superhologram. The large transform lens acts as part of a 4f relay to scale, transfer, and angularly tile the elemental holographic patterns to the superhologram image plane. The outer zones of the large transform lens must redirect the light and views into the wider views of the hologram image in front of the lens.

#### Holobricks

**Fig. 1:**
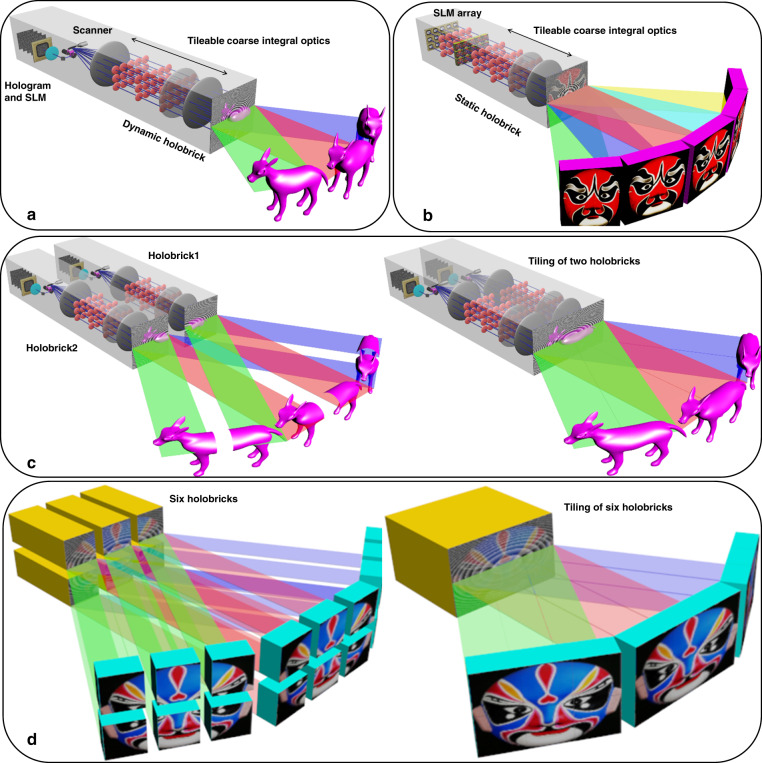
Schematic of tileable holobricks. **a** Dynamic holobrick structure. **b** Static holobrick structure. **c** The spatial tiling of two dynamic holobricks. **d** The spatial tiling of six static/dynamic holobricks to display a large object

We design CIH holobricks (complete CIH system modules) that can be seamlessly tiled together to form a large holographic display wall. Figure 1 shows the schematic of the spatially tileable modular CIH display structure. Solid-state and scanned holobricks shown in Figures (a) and (b) are realized by a tileable CIH display mechanism. Similar to the original CIH displays, the modular CIH adopts coarse integral optics to allow angularly tiling of multiple elemental scaled holograms; however, they are modified so each holographic pattern is now able to fill the entire face of a holobrick allowing the modules to be spatially abutted to form the montage. As shown in Fig. 1c, d, multiple holobricks can be spatially tiled, which is capable of appending multiple wide-viewing-angle and small-size holograms into a single larger size mosaiced hologram with the same wide FOV. Our previous CIH methods shown in Supplementary Fig. S1 utilized the CIH to implement the angular tiling of holograms to improve the FOVs in a single direction. They are not tileable and cannot simultaneously increase the image size and viewing angle because their non-tileable nature limits the scaling capability of the CIH display’s SBPW. The holobrick display structure shown in Fig. 1 is spatially tileable, which can overcome the limitations of the previous CIH methods for a high SBPW display and has the capability of increasing image sizes and viewing angles simultaneously in both directions.

#### Solid state holobricks

A hologram array provides the modular coarse integral hologram model to construct a solid-state holobrick display system. In this hologram array, each subhologram includes complete 3D information (depth, parallax, etc.) and provides a specific small-viewing angle. In the solid-state holobrick, the hologram array is attached to the tileable coarse integral optics configured by an array of scaled offset periscopes. The solid-state holobrick system still can be configured separately and also flexibly allocated FOV information between the horizontal and vertical direction.

Holobricks include solid-state and scanned (dynamic) versions based on the sCIH and dCIH configurations. Each holobrick uses the array of scaling offset periscopes as modular coarse integral optics capable of angularly tiling the multiple narrow-FOV scaled holograms into a single wide-FOV scaled superhologram (matching with the optics size). Multiple holobricks spatially append multiple wide FOV and small-size holograms into a single larger size mosaiced hologram with the same wide FOV.

To form a solid-state holobrick, an array of DMDs are relayed to the input lenslet array of the scaling offset periscopes that acts as a tillable coarse integral optic creating the angularly tiled and scaled relayed super hologram. The field lenslet array keeps the rays confined to their bundles. Then a large common transform lens in contact with the relay lens array redirects and angularly tiles the elemental holograms into a super hologram at the field lens. The field lens redirects the outwardly splayed view-angles to be symmetric about the module’s surface normal. For very large systems, offset field lenses or prisms may be used to create modules with asymmetric fields of view to direct those modules’ views into a common view zone or headbox, ensuring the module’s hologram is seen from the viewer’s locations.

The solid-state holobrick layout is shown in Supplementary Fig. S3. A scalable non-scanning system can be formed by multiple solid-state holobricks with spatially tiled holographic images, as more and more holobricks may be added to benefit the gain of the displaying area size of the reconstructed holographic images. Therefore, the scalable CIH system (such as holographic wall) with multiple holobricks enables ultra-large size and large FOV holographic display in both directions due to spatially tiled holographic images. An example of the spatial tiling using two and three static holobricks with holographic images is shown in Supplementary Figs. S4–S6.

#### Scanned holobricks

To decrease the number of SLMs in each holobrick, while still providing sufficient space-bandwidth product, the hologram array input of the modular CIH display can also be implemented by scanning a high-BW SLM to create the elemental hologram pattern and image array behind the coarse integral periscope array optics. Just like the coarse integral optics in the holobrick, the scanning sub-system cannot be larger than the final holographic image as this would prevent tiling of the holobricks. The optical architecture of a standard scanning system is also often a 4f relay with the scanner at the Fourier plane. In the standard scanning system, the large output transform lens is much bigger than each scanned image, because it needs to redirect all the light and provide views of those images.

To make a modular scanning system, we can similarly replace the 4f relay with a periscopic relay to make the scanned elemental hologram and scanning system the same size, with a field lens at the SLM, a scanner at its focal plane, a large transform relay lens, and a field lens array at the elemental image plane collocated with the input to the modular coarse integral optics. With a slight modification, we can also space the DMD from the front transform lens, and have the scanned DMD image appear behind the scanned periscopic relay’s final lens array but in front of the modular coarse integral optics entrance field lens array. With a virtual attached lens computed into the hologram, this configuration is creating a holographic image on the scanner, and scanning an array of 3D holographic images at the entrance of the modular coarse integral optic to angularly tile.

**Fig. 2:**
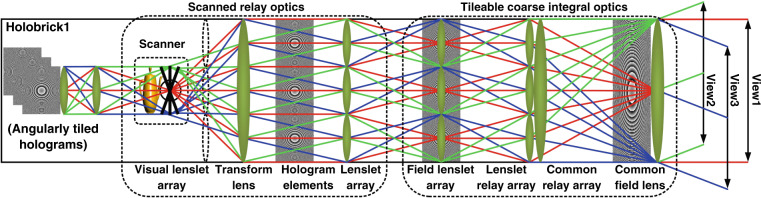
The schematic of a scanned holobrick with the scanned modular CIH

Figure 2 shows the schematic of scanned holobricks with the scanned modular CIH. The scanned modular coarse integral periscope array optics is a scanned offset periscope consisting of a scanned relay optics and modular coarse integral optics.

In a solid-state or scanned holobrick, the width or height of a relayed subhologram from the SLM array or scanners is equal to that of its corresponding lenslet in order to implement the gap-free and overlap-free tiled viewzones. An example of the holographic image layout of a scanned holobrick is shown in Supplementary Fig. S7.

**Fig. 3:**
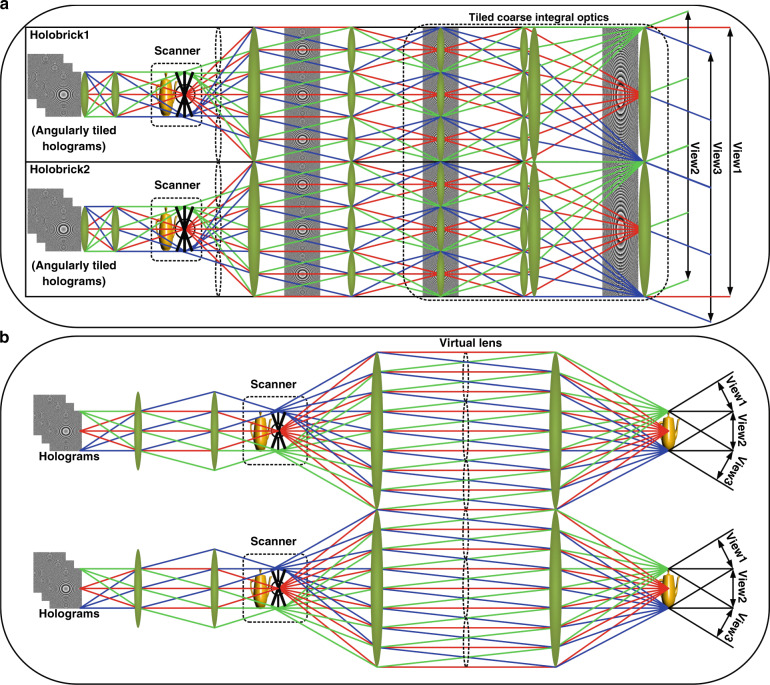
Schematic of scanning CIH using modular coarse integral optics for spatial tiling of scanned holobricks. **a** Two scanned modular CIHs in two scanned holobricks spatially tiled to achieve the same FOV angle but the doubled size of the reconstructed holographic image, **b** the previous scanning sub-system in the complete bandwidth-utilization CIH configuration that is not tileable

Figure 3a shows the schematic of the modular coarse integral hologram display structure. The modular CIH display structure is composed of an array of holographic bricks, with each brick angularly tiling elemental holographic patterns using offset periscopic coarse integral optics constituting a field lenslet array, a relay lenslet array, a large common transform lens, and a large common field lens. The field lenslet array and large common field lens are arranged at the front focus plane position of the relay lenslet array and the large common transform lens (the spacing between them is set to zero), forming an array of tiled offset scaled periscope relay system. The angularly tiled sub-holograms and elemental holographic images are attached to the front focus plane position of the relay lenslet array. The scaled hologram plane is relayed to the front focus plane position of the large common transform lens. As shown in Fig. 3b, it is noted that our previous scanning sub-system in the complete bandwidth-utilization CIH configuration is not tileable and hence cannot be spatially tiled.

Both the scanning and coarse integral optics subsystems both use periscopic optics. The scanned periscopic relay optics create a smaller elemental hologram but larger viewing angles. The scanned area can be as large as the transform lens. Then, the modular coarse integral optics can scale and angularly tile scanned hologram matching with the large transform lens. With the scanning and coarse integral optics subsystems’ elemental holograms, lenses and super-hologram being the same size allow us to seamlessly abut and spatially tile multiple hologram arrays. An example of the spatial tiling using three dynamic holobricks with holographic images is shown in Supplementary Figs. S8 and S9.

### Experiments

We experimentally demonstrate a modular holographic display with two holobricks. We describe the setup, the synchronization, the design and fabrication of the periscope optics, and also the computer-generated hologram rendering. We then display 2D holographic images for calibration, 3D holographic stereograms of physical objects, and finally 3D holograms of computer-generated objects. In these experiments, the holobricks are not optimized to maximize the use of the scanner, modulator, or optics, but rather to demonstrate the spatial tiling ability of multiple holobricks.

#### Experimental setup

**Fig. 4:**
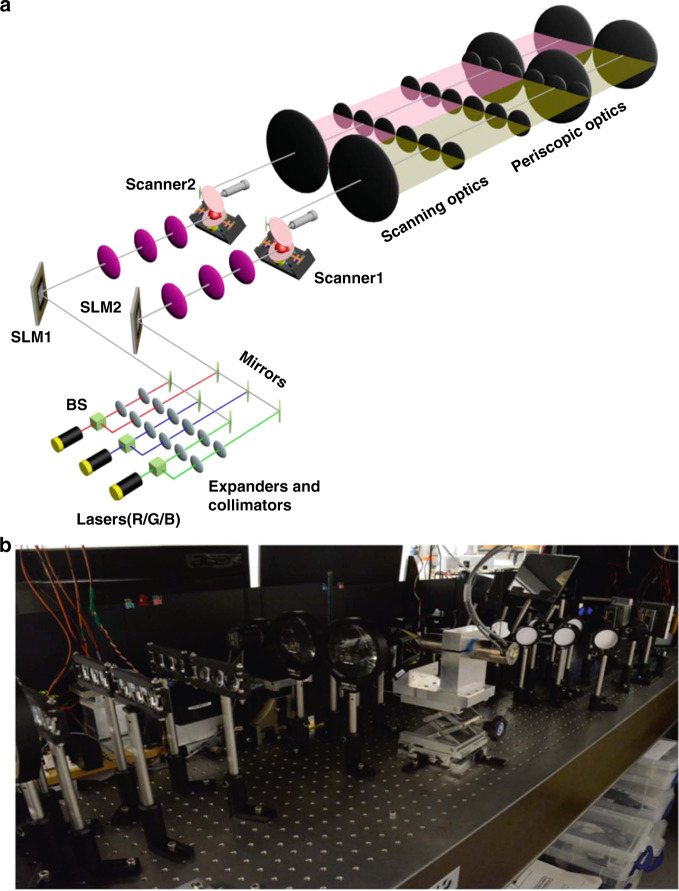
A proof-of-concept system of two holobricks. **a** The schematic setup, and **b** the experimental setup

A sketch of the proof-of-concept system is shown in Fig. 4 and Supplementary Fig. S11. Two same DMDs are used as the MEMS-SLMs of two holobricks. Each DMD with 1024 × 768 pixels consists of a DLP v4100 controller and TI 4100 chipset, which is the same module as for the previous scanned full-bandwidth CIH system. The DMD’s pixel pitches are 13.7 μm × 13.7 μm horizontally and vertically. Thus, the available active area of the DMD is 14 mm × 10.5 mm. The pattern frequencies of the DMD can achieve 22,272 Hz global array updates per second for a frame period of 44.89 µs. The full bandwidth of each of the DMD is 17.5 Gbit/s, thus providing the full bandwidth of 35 Gb s^−1^ in a holobrick system. The high-BW DMD of each holobrick is sequentially illuminated by red (660 nm), green (532 nm), and blue (450 nm) lasers. The three lasers all have a modulation frequency of 150 MHz. The holobrick uses a resonant scanner (SC-21) and a galvanometric scanner (6260HM44A) to implement the angular tiling of holograms in the dynamic holobricks. The resonant scanner has a scanning angle of 40°, which is also the FOV of a holobrick. We also designed a color-beam-combination optical structure composing of three self-governed optical channels capable of six-degrees-of-freedom control to implement the complete combination of three color beams modulated by a DMD. The SBWP of two tiled holobricks can achieve 142.2 × 10^9^ bit s^−1^. Accordingly, the optical extent of two tiled holobricks is 5.2 × 10^6^ mm^2^ deg^2^ s^−1^.

#### Synchronization controlling of holobricks

To manage multiple holobricks, the synchronization controller was designed to manage all devices involving lasers, DMDs, and optical scanners. The synchronization controller was exploited on a Labview software platform, where the software version is LabVIEW 2013 from the National Instruments (NI). The connector between the synchronization controller and holographic display system is a multi-function DAQ card (USB-6341, X Series DAQ) with two analog-output channels and a maximum sample rate of 500 kS s^−1^. In the synchronization controller, we designed a repeatable trigger mode to generate the synchronous controlling signals for holobricks. This controller has two sub-controllers with the same control configuration for each holobrick’ DMD with different identification addresses. It configured the DMD into a frame output sync mode and slave mode to synchronously control the lasers at the hardware level, which also adopted the same trigger signals to synchronously control two holoricks’ DMDs for displaying tiled holograms. Moreover, this controller sets into an infinite loop mode to fully utilize the DMD information bandwidth and to produce a continuous holographic video sequence as well. To efficiently manage scanners of holobricks, we also exploited a boustrophedon scanning pattern method to synchronously control the galvanometric-based and resonant-based optical scanners. The synchronization working of all DMDs, lasers, and scanners using this controller can ensure the successful implementation of wide-viewing-angle and large-size holographic image displays capable of the full-bandwidth utilization of all SLMs in the spatial tiled holobricks.

#### Design and fabrication of the array of offset scaled periscope optics with bundle rays

An array of offset scaled periscope optics is an important sub-system that is utilized to construct the optical systems of holobricks with modularity in this paper. The periscope optical array is constructed by transforming the 4*f* optical structure to spatially tile multiple CIH systems without seams, enabling ultra-large size display. The fabrication process of an array of offset scaled periscope optics is shown in Supplementary Fig. S12. The periscope optics is formed by the 4f relay lenses in connection with field lenses. The periscope optics can keep the rays confined to their bundles, which have the smallest tube diameter compared to the other 4f relays. Then, the double lens structure replaces the relay 2*f*-lens in the periscope system to form the offset periscope optics structure. The array of the scaled offset periscope optics is achieved by spatial tiling of sub-lenses to seamlessly abut multiple 4*f* relay arrays. A large lens in contact with the relay lens array redirects and combines the array outputs into a super-hologram the size of the array. A final field lens at the holographic image plane bends the output views to make the system afocal telecentric. The offset scaled periscope configuration is applied to the sCIH and dCIH to form the solid-state and scanned holobricks.

#### Experiment procedure and results

##### 2D holographic images (calibration)

**Fig. 5:**
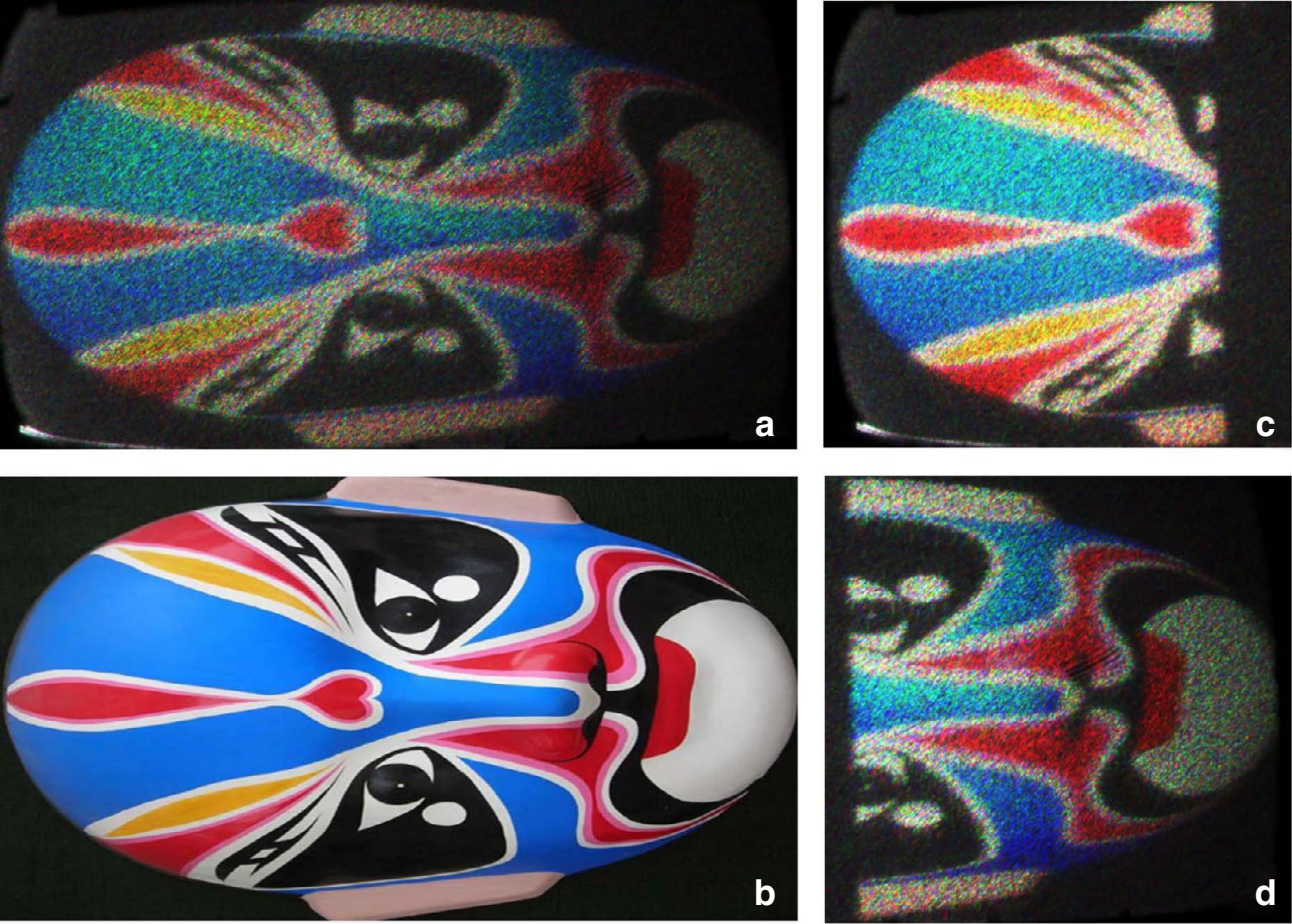
Holographic display results of the two spatially tiled holobricks. **a** Reconstructed holographic images with two holobricks, **b** the original image, **c** reconstructed holographic images of first holobrick, and **d** reconstructed holographic images of second holobrick. Each holobrick displays a half part of the whole object and two holobricks display a large-size holographic image

A Peking opera mask is used as the test object to calibrate two holobricks when implementing the holographic stitching. Our CGH generation algorithm is adopted to calculate the Fresnel holograms, which can be reconstructed without a Fourier lens. The calculated holograms are presented on the DMDs of the two holobricks. Two holobricks are spatially tiled in the horizontal direction. Three colors (R/G/B) have also been embedded into the two holobricks by using view sequential color and exploiting the SLM’s high-BW property. Figure 5 demonstrates the holographic display results of the two spatial tiled holobricks. Each holobrick displays a half part of the holographic image of the large opera mask object. Other reconstructed holographic image results from scanned holobricks are shown in Supplementary Materials. When two holobricks are not matched due to assembly error or beam mismatching, the image quality at the edges decreases, which may result in some artifacts that are not accepted by viewers. When this case happens, a pre-compensation calibration method can be used to improve the quality of the whole large display. Firstly, the artifacts of the edges of the display system are measured. Then, the artifacts are embedded into the hologram generation stage. Based on this calibration, we found that the display can obtain a good display quality accepted by an observer.

##### 3D holographic stereogram of physical objects

**Fig. 6:**
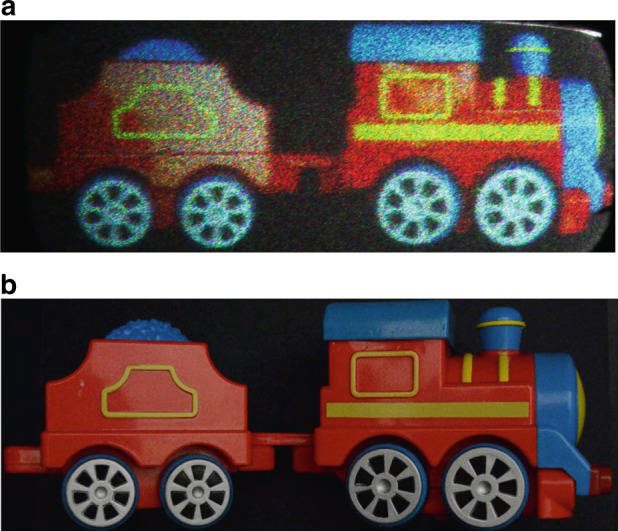
Reconstructed holographic images of a toy train with holobricks. **a** Reconstructed holographic images, and **b** original image captured by a camera

Photographs of a toy train composed of a locomotive engine and a tender coal car are used to generate and display the spatially tiled holographic stereogram image on two tiled holobricks. An actual camera (Nikon D7000) is utilized to acquire the original color images of the toy train and the captured images are used to calculate the Fourier holograms. The generated holograms are presented on the two holobricks. Figure 6 shows reconstructed holographic images of a toy train with two tiled holobricks. The engine of the toy train is displayed by the first holobrick and the tender car of the toy train is displayed by the second holobrick. Each holobrick displays a half of the whole toy train and two holobricks display a large size holographic image.

##### 3D holograms of computer generated objects

We used a 3D object model to perform the holographic display experiments for the two dynamic holobricks. The Autodesk 3DS Max platform (64 bit, 2012 version) is utilized to produce different image parallax views of the 3D object model. The holographic diffraction patterns of different parallax views and different spatial areas can be generated by CGH algorithms^37–39^. We previously develop multiview-multilayer hologram rendering algorithms compatible with our holographic video system^40,41^. The layer-based CGH provides accommodation cues, while occlusion/disocclusion effects are updated for each angularly tiled sub-hologram. Accordingly, here, we utilize our CGH algorithm^42^ to calculate different parallax sub-holograms for scanned holobricks. Although the holobricks are compatible with our multiview multilayer-based CGH algorithm to achieve occlusion/disocclusion and accommodation cues; for simplicity of this experiment, we only demonstrate an occlusion capable multiview hologram but do not include accommodation cues. The detailed hologram generation algorithm is shown in Supplementary S2. The generated holograms are reconstructed and displayed with the two tiled holobricks. The tiled holobricks can deliver different spatial sub-holograms and parallax sub-holograms to their targeted spatial array positions and the desired FOV angle.

**Fig. 7:**
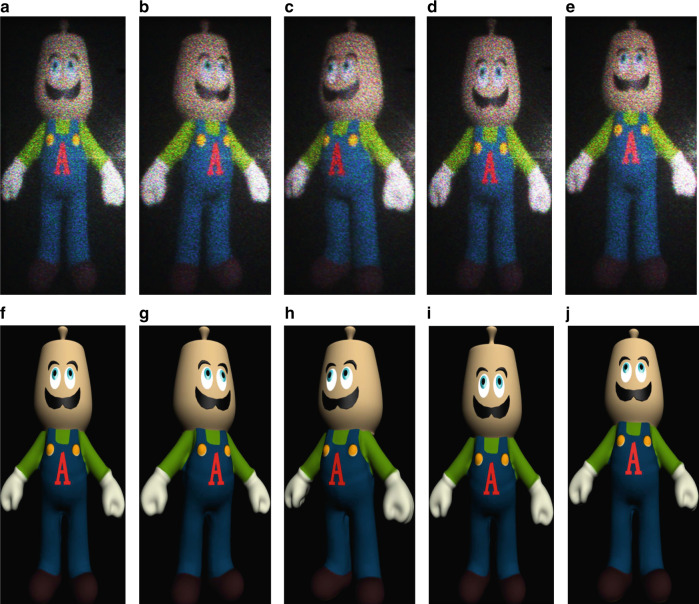
Reconstructed holographic images of a 3D teapot man using dynamic holobricks at different viewing angles. **a** Results of the 0° position, **b**, **c** images of −20° and +20° in the horizontal direction, **d**, **e** displaying at −3° and +3° in the vertical direction, and **f**–**j** original images at different viewing angles at 0°, −20°(horizontal), +20°(horizontal), −3° (vertical), +3° (vertical), respectively

Figure 7 demonstrates the reconstructed holograms of a spatial tiled 3D teapot man through the proposed tiled holobricks at different FOV angles. Each holobrick displays half of the holographic fringe pattern at different viewing angles. We also use a camera to capture different viewing-angle images of two 3D toy persons to perform the display experiment results. Reconstructed holographic images using real 3D objects are shown in Supplementary Figs. S14 and S15.

## Discussion

These results indicate that the proposed holobricks can display a 2D holographic image, a 3D holographic stereogram, and a full 3D holographic image with the scalability of image area size and FOV angle compared with the utilization of the SLM only. Although we have shown 3D stereograms (no accommodation cues) captured from physical objects, and full 3D holograms of using CG renderings, we can of course, use a depth plus color camera (dRGB), e.g. Intel depthSense camera or Microsoft Kinect, to create a multilayered Fresnel hologram, with associated accommodation cues, from multiple dRGB images of a physical object.

Compared with a single holobrick, two holobricks can display a holographic image of twice the width due to the use of all-optical spatial tiling of two holobricks, where each holobrick contributed a halved part of the full area of the final reconstructed holographic image. The experimental results indicated that the seamless mosaic of two tiled holobricks can be achieved by the modular coarse integral optics.

In the experiment, we also found that the proposed holobrick display can reconstruct the detailed surface brightness of the original images that are disturbed by the external imaging conditions. When the reflected or scattered light of a 3D object (LED illumination) is acquired by the experimental camera (Nikon D7000), the imaging process is a complex link involving the light illumination uniform, the surface reflection nonlinearity of the object, imaging link blur (point-spread function, image sensor, exposure times, etc.), etc. The captured images are different at different exposure times. The proposed tiled holobricks can display the differences for the same object. Reconstructed holographic image results when the original images are acquired under various exposure intensities are also exhibited in Supplementary Fig. S16. It can be seen that when the surface brightness of the original image is not uniform, the displayed image also can demonstrate these differences. This indicated that the proposed holobrick display has a high display performance.

In conclusion, we proposed a proof-of-concept of holobricks with tileability for achieving 3D holographic displays endowed with the ultra-large size and large viewing angle. A holobrick is formed by a static or dynamic tileable CIH based on tileable coarse integral optics (i.e., arrays of offset scaled periscope) that replay the hologram array and angularly tile them to attain a large-viewing-angle display. Our previous research in scalable CIH displays is compatible with holobricks, allowing us to incorporate multiple SLMs and multiple higher performance scanners to increase the field of view, size, and information content of each holobrick. All-optical spatial tiling of multiple holobricks is operated to form a modular holographic display system enabling the simultaneous realization of a large-size and wide FOV.

Apart from the capability of scaling up by incorporating more holobricks to gain dynamic ultra-large-size wide-FOV 3D holographic images, the scalable holographic display system can tile holobricks into a malleable holographic display configuration, which may lead to advances in different holographic display tasks (e.g., holographic walls). We expect that this work paves a new promising way for ultra-large-size and large-viewing-angle holographic displays with the currently limited display capability of SLMs. These holobricks will be able to enable a wide range of applications for their capability of tiling together in a flexible display format. Based on the customer application requirements, they can be designed into different potential holographic display products, such as holographic video displays, holographic video walls, holographic interactive kiosks, etc.

## Materials and methods

### Coarse integral periscopic array

To seamlessly abut multiple hologram relay arrays, we must modify the relay in the CIH architecture so the lenses are not larger than the holograms’ raybundles in the system. We note periscope optics (field plus relay lenses) have the smallest “tube diameter“, compared to the other relay optics (e.g. 4f relays). In the basic periscope, the object, lenses, and image are all the same size and the ray bundles all fit in the tube. Basic periscope relays are spatially tileable (each with inverted images), but they do not angularly tile their objects at the image plane. We need a coarse integral optics equivalent of the periscope – an optical system that angularly tiles an array of elemental holograms/images to the super hologram that fills the entire output aperture so multiple systems can be seamlessly spatially tiled into an array.

**Fig. 8:**
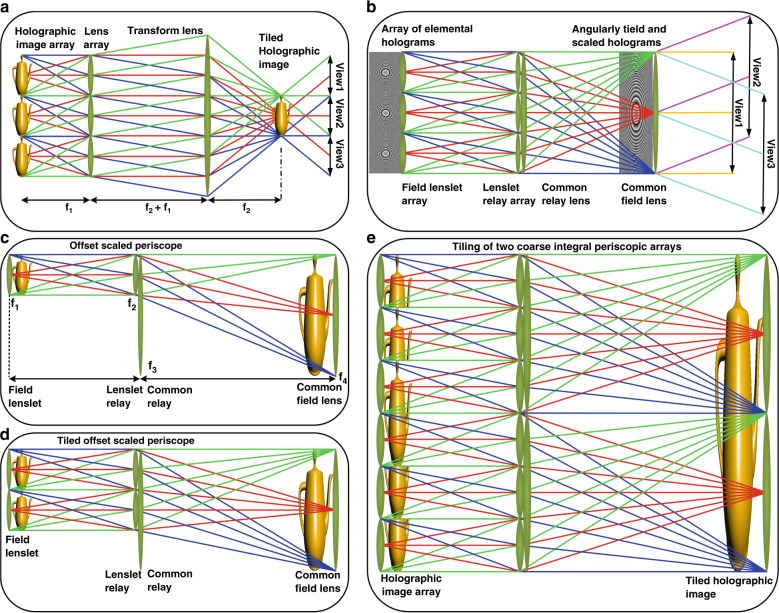
Offset scaled periscope structures. **a** A standard 4f relay in the original CIH where the holographic image is smaller than the display optical system, **b** schematic illustration of the tileable CIH using an array of tiled offset scaled periscope optics, **c** offset scaled periscope optics, **d** two tiled offset scaled periscopes, and **e** spatial tiling of two coarse integral periscope optical arrays

We modify the standard periscope relay by splitting the relay into a large transform lens and an offset lenslet to form an offset scaled periscope, as shown in Fig. 8. A standard 4f relay in the original CIH display in Fig. 8a can only provide a holographic image that is smaller than the display optical system. We constructed a tileable CIH using an array of tiled offset scaled periscope optics shown in Fig. 8b, which is capable of simultaneously generating angularly tiled and scaled holographic images with the same size as the optics. The offset periscope relay as shown in Fig. 8c, d can create the offset and skewed holographic image, but the image size is still the same as the object size. A scaling offset periscope relay can be obtained by changing relay lenses powers to demagnify the relayed hologram. Each relayed elemental hologram is enlarged to be same size as the array optics but a proportionally reduced field of view. As shown in Fig. 8e, multiples of the offset scaled periscope optics can be spatially tiled to form a large holographic image with the same FOV as the single one. The transform process from the plain periscope to the array of offset scaled periscope array is shown in Supplementary Fig. S2.

### Analysis

To understand the magnitude of improvements the modular CIH system provides, we analyze the FOV, the image size, space-bandwidth product, bandwidth, of tiled CIH systems and the optical extent of the holograms they produce.

#### FOV of tiled holobricks

Multiple holobricks are spatially tiled to form a modular coarse integral hologram display. A holobrick has a specific FOV angle formed by the modular scanned CIH. The view angle of a holobrick can be calculated by:

6 $${\mathrm{FOV}} = {\mathrm{FOV}}_x \times {\mathrm{FOV}}_y = \theta _x \times V_y \times \theta _x \times V_y$$

where *θ*_*x*_ and *θ*_*y*_ are the horizontal and vertical viewing zone of each subhologram, *V*_*x*_ and *V*_*y*_ are the total horizontal and vertical FOV numbers created by the holobricks’ 2D scanner, respectively. In the horizontal and vertical direction, the diffraction FOV zone (denoted by *θ*_*x*_ and *θ*_*y*_) of a sub-hologram presented on the holobrick’ SLM is calculated by:

7 $$\sin \theta _x = \frac{{m\lambda }}{{2d_x}},\,\sin \theta _y = \frac{{m\lambda }}{{2d_y}}$$

where *m* denotes the diffraction order of subholograms, λ is the wavelength of the laser illumination beam, the *d*_*x*_ and *d*_*y*_ express a horizontal and vertical pixel pitches of the holobricks’ SLM, respectively.

Since the SLM pixel pitch is very small, there are sin*θ* ≈ tan*θ* ≈ *θ*. The total viewing angle of a holobrick can be expressed as:

8 $$\begin{array}{l}{\mathrm{FOV}} = \sin ^{ - 1}\left( {\frac{{m\lambda }}{{2d_x}}} \right) \times V_x \times \sin ^{ - 1}\left( {\frac{{m\lambda }}{{2d_y}}} \right)\\ \qquad\quad\,\, \times\, V_y \approx \frac{{m^2\lambda ^2}}{{4d_xd_y}} \times V_x \times V_y\end{array}$$

For the tileable periscope system, the total viewing angle of a holobrick can be expressed as:

9 $${\mathrm{FOV}} = \frac{{f_1}}{{f_2}}\frac{{m^2\lambda ^2}}{{4d_xd_y}} \times V_x \times V_y=\frac{1}{\beta }\frac{{m^2\lambda ^2}}{{4d_xd_y}} \times V_x \times V_y$$

where *f*_1_ is the FL of the lenslet in the relay lenslet array of the tileable coarse integral optics, *f*_2_ is the FL of the large common transform lens in the modular coarse integral optics, *β* = *f*_2_/*f*_1_ (*f*_2_ > *f*_1_) is the scaling factor of the offset scaled periscope. The tiled holobricks’ viewing zone can be expressed as

10 $${\mathrm{FOV}}^\prime = \frac{1}{\beta }\frac{{m^{2}m^{2}}}{{4d_{x}^{\prime} d_{y}^{\prime} }} \times V_{x}^{\prime} \times V_{y}^{\prime}$$

where *V*_*x*_ = *N*_*x*_ × *V*_*x,*_*V*_*y*_ = *N*_*y*_ × *V*_*y*_, $d^{\prime}_{x}$ = *N*_*x*_ × *d*_*x,*_$d^{\prime}_{y}$ = *N*_*y*_ × *d*_*y*_, *N*_*x*_ and *N*_*y*_ represent the total tiling number of holobricks in the horizontal and vertical direction, respectively.

Based on Eq. (9) and Eq. (10), FOV′ = FOV. This indicated that the modular CIH has the same FOV as the single holobrick. At each FOV angle, the holographic image of tiled holobricks is spatially tiled. In our experiments, FOV′ = FOV = 40°. Thus, each holobrick (or the tiled holobircks) has a viewing angle of 40°. The spatial tiling of two and three holobricks and holographic image arrangement of each FOV are also shown in Supplementary Fig. S10.

#### Holographic image size of tiled holobriks

In a solid-state or scanned holobrick, the numerical aperture of a relayed sub-hologram from the SLM array or scanners matches that of its corresponding lenlset, enabling the seamless abutment of tiled viewzones. The reconstructed holographic image size is expressed by

11 $$L = 2f_1\tan \left( {\frac{1}{2}\theta } \right) = 2f_1\tan \left( {\frac{1}{2}\sin ^{ - 1}\left(\frac{{m\lambda }}{{2d}}\right)} \right) \approx f_1\frac{{m\lambda }}{{2d}}$$

where *L* is the width or height of the holographic image (i.e., the diameter of a lenslet). *f*_1_ is the lenslet’s FL in the relay lenslet array of the modular coarse integral optics. For the offset scaled periscope, the scaled hologram can be expressed as:

12 $$L^\prime = \frac{{f_2}}{{f_1}}L = \beta L$$

where *f*_2_ is the FL of the large common transform lens in the modular coarse integral optics, *β* is the scaling factor of the offset scaled periscope.

Let the tiling number of holobricks be *N*. The tiled holobrick image size is equal to *L* = *L*′ × *N*. From Eq. (9) and Eq. (12), the sub-hologram size magnification scale (i.e., *β* > 1) due to the scaled periscope system is inversely proportional to the demagnification (i.e., 1/*β*) of the scaled hologram’s FOV angle. The scaled hologram size is the *M* times greater than the sub-hologram size. The total viewing angle of the scaled hologram is the *V*_*x*_ or *V*_*y*_ time greater than the demagnified diffraction angle of sub-hologams.

#### Hologram display capability of holobricks

We analyze three aspects of the hologram display’s capabilities for holobricks and the holographic images they produce: information bandwidth, space bandwidth products, and optical extent. The SBWP of the modular holobricks can be calculated by

13 $${\mathrm{SBP}} = \frac{{W \times H}}{{a \times b}} \times V_x \times V_y \times F \times S \times N$$

where *N* denotes the total number of holobricks in the holographic display, *W* and *H* express the vertical and horizontal size of the hologram, *a* and *b* represent the vertical and horizontal pixel pitches of the holobricks’ SLM, *S* denotes the color-component’s number (holobricks’ lasers), *F* represents the displayed holographic-video frame rate, *V*_*x*_ is the total horizontal FOV number of tiled holobricks, and *V*_*y*_ is the total vertical FOV number of tiled holobricks.

For example, using our previous system’s’ specifications, the SBP of dCIH is equal to SBP = 36.0 × 10^9^ patterns s^−1^. Accordingly, the full bandwidth CIH can achieve approximately the bandwidth of 71.2 × 10^9^ bit s^−1^. The modular CIH with tiled holobricks can obtain the bandwidth of *N* × 71.2 × 10^9^ bit s^−1^. For example, when *N* = 2, the bandwidth of the holobricks is 142.2 × 10^9^ bit s^−1^.

The optical extent of the holographic images produced by the tiled holobricks can be expressed as:

14 $$A\Omega = W \times H \times \Delta \Phi \times \Delta {\uppsi} \times V_x \times V_y \times F \times S \times N$$

where *N* is the total number of the tiled holobricks, *W* and *H* denote the horizontal and vertical size of a sub-hologram, *S* represents the lasers’ number (holographic video colors), Δ*Φ* and Δ*Ψ* are the horizontal and vertical view zone of each sub-hologram, *F* represents the frame rate of the holographic video reconstructed by the tiled holobricks.

The dCIH system displayed a hologram using an SLM of 13 mm × 10 mm with an optical extent calculated to be AΩ = 1.3 × 10^6^ mm^2^ deg^2^ s^−1^. The full-bandwidth dCIH system can display the optical extent of 2.6 × 106 mm^2^ deg^2^ s^−1^. The optical extent of a tiled holobricks version would be N × 2.6 × 10^6^ mm^2^ deg^2^ s^−1^. For example, a scalable CIH system using six tiled holobricks (*N* = 6), the optical extent is 1.5 × 10^7^ mm^2^ deg^2^ s^−1^. The modular holobricks can overcome the capability limit of current hologram 3D displays.

In summary, the field of view of every holobrick is the same, but the spatial tiling of the modules increases the size proportionally. The overall bandwidth and space-bandwidth product of the CIH system increase linearly, while the optical extent of the holograms produce also increase linearly with the number of holobricks.

## Supplementary information

Supplemental material

Supplementary video
