# Correction to: Holobricks: modular coarse integral holographic displays

**DOI:** 10.1038/s41377-022-00771-2

**Published:** 2022-03-31

**Authors:** Jin Li, Quinn Smithwick, Daping Chu

**Affiliations:** 1 Centre for Photonic Devices and Sensors, University of Cambridge, 9 JJ Thomson Avenue, Cambridge, CB3 0FA UK; 2 Disney Research, 521 Circle 7, Glendale, CA 91201 USA

**Keywords:** Displays, Integrated optics

Correction to: *Light: Science & Applications*

10.1038/s41377-022-00742-7 published online 16 March 2022

Following publication of this article, it is noticed that Fig. 1 contained some typos. The correct figure is published in this Correction.^1^
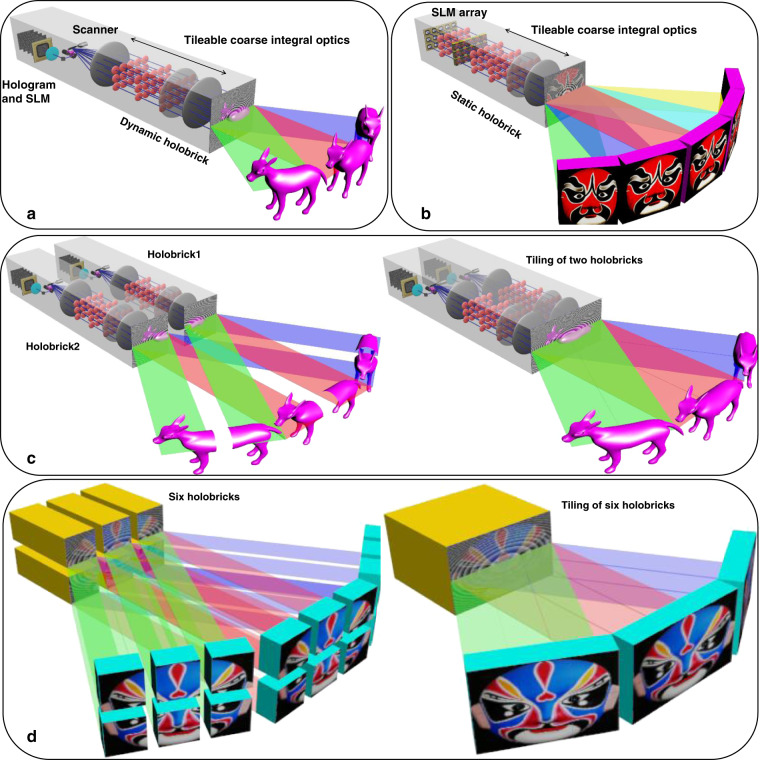


The original article has been updated.
