# Supplementary material for: Holobricks: modular coarse integral holographic displays
Source: Light Sci Appl. 2022 Mar 16;11:57. doi: 10.1038/s41377-022-00742-7 (PMC8924222; doi:10.1038/s41377-022-00742-7)
Supplement: Supplementary file 1 — Supplemental material [file 41377_2022_742_MOESM1_ESM.pdf]

Supplementary information for
Holobricks: Modular Coarse Integral Holographic Displays

Jin Li[1] , Quinn Smithwick[2] , Daping Chu[1,*]

[1]*Centre for Photonic Devices and Sensors, University of Cambridge, 9 JJ Thomson Avenue, Cambridge, CB3 0FA, UK*
[2]*Disney Research, 521 Circle 7, Glendale, California 91201, USA*

[*]Corresponding author: dpc31@cam.ac.uk

S1. Previous Coarse Integral Holography

A. Solid-state and dynamic Coarse Integral Holography

The Coarse Integral Holographic (CIH) display forms a wide-viewing-angle holographic display (i.e., higher optical invariant etendue) by utilizing Coarse Integral Optics (CIO) to implement angularly tiling of the fields-of-view of multiple low optical extent holograms generated by sweeping a small space-bandwidth product (SBWP) but large bandwidth (BW) (e.g., high kHz pattern rates) SLM. Figure S1 exhibits an overview of the previous CIH principle framework.

In the solid-state CIH (or sCIH) display adopted an array of low SBWP SLMs is attached to the CIO constituting an appropriate lenslet array and an integral transform lens. Note that the term "solid-state" refers to the "static" or "non-scanning" in this paper. Each SLM creates its own low optical extent hologram capable of presenting complete 3D full-parallax holographic images but representing a slightly different small view-zone, The coarse integral optics act as an array of progressively offset optical relays, angularly-tiling the many low optical extent holograms into a single "super-hologram" with huge etendue. The sCIH can flexibly select the array layout to separately adjust field-of-view (FOV) information in the horizontal and vertical direction.

Furthermore, a scanned or dynamic CIH (dCIH) display was developed by sweeping a single high-BW SLM to create the hologram pattern array for the CIO at video frame rates [1]. To create holographic video, the array of holograms must be laid out within the period of one holographic video frame. However due to the scanner's limited practical sweep angle and mirror size, the scanning system could not seamlessly distribute all low optical extent holograms produced by a SLM at full BW condition. The galvanometer was the bottleneck in using the full bandwidth of

the system. In other words, the information BW controlled by the galvanometer (the product of the mirror area size, scan angle, and sweep frequency) was still less than the information bandwidth SLMs was capable of to produce the super-holograms that at video frame rates. Even after allowing overlapping holograms with independent color components for view sequential color, the early dCIH displays can only implemented the SLM’S bandwidth utilization ratio of 53% with the available scanners at the time.

B. Scalable Coarse Integral Holography

To address this issue, various scanning mechanisms were explored that permit better complete utilization of the SLM’s BW in the CIH display. The unabridged SLM’s bandwidth utilization enabled the CIH display to produce larger, wider FOV holographic images at video frame rates. We investigated three different scanning configurations to increase the bandwidth of the scanning system: two utilizing resonant scanners, with the large mirrors, large deflections, and high but fixed scan frequencies, and a third setup using multiple scanners.

In the first setup, the dCIH added high-frequency resonant scanning in the kHz range as a high-speed vertical dither [2]. This resonant scanner’s scan frequency was too high to use for line rate scanning of the horizontal array of holograms to seamlessly abut without gaps. It was therefore used as an auxiliary dither scanner to add a vertical zig-zag dither to the existing horizontal scan, thereby tiling a few holograms in a vertical column for every horizontal row tiling. This arrangement could not increase the number of horizontal hologram tiles, but rather increased the number of vertical tiles, and as a result, increased the display’s vertical FOV, and vertical headbox size.

In a second setup, the unutilized SLM bandwidth was exploited by using the dCIH display

configuration but replaced the horizontal galvanometer scanner and mirror with a taut-band-based resonant optical scanner (at the identical scanning frequency of 70K Hz) and a larger-size mirror [3]. The exploitation of the wide-angle and large-mirror resonant optical scanner benefited the two-fold increase of the horizontal FOV angle and employed the entire BW of the SLM.

Finally in a third setup, two synchronous scanners were cascaded to enlarge the horizontal viewing angle of reconstructed 3D holographic images and to fully exploit the information BW of the SLM [4].

Using any one of these schemes, the scanners had more than sufficient bandwidth to handle the information in the holograms produced by the SLMs. The scanners were no longer the bottleneck in the system. To further expand the optical extent of the holograms created by the CIH system, another setup used two full-bandwidth holograms produced on two high bandwidth SLMs. These SLMs spatially tiled onto and scanned by a common large-area taut-band-resonant-based optical scanner (horizontal) and a large-size galvanometric-based scanner (vertical). Then, they angularly tiled by a large integral lens [5]. This results in a full bandwidth dCIH display capable of allowing the two-fold reconstructed holographic image size and twice the viewing angle in the horizontal direction at the same time.

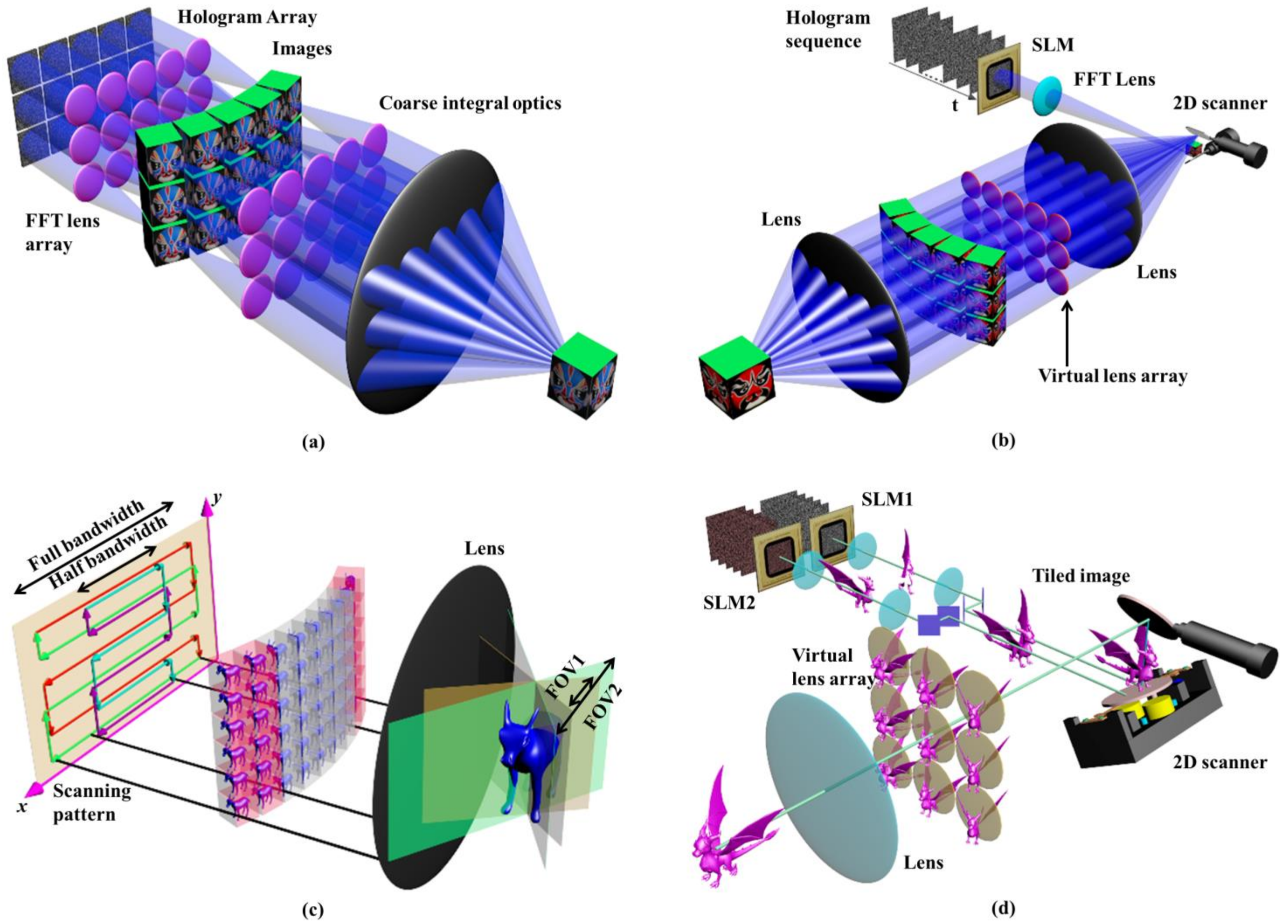

Fig. S1. Review of the CIH framework, (a) sCIH structure, (b) dCIH configuration, (c) full-BW dCIH principle, and (d) schematic of image spatial tiling of full-BW dCIH.

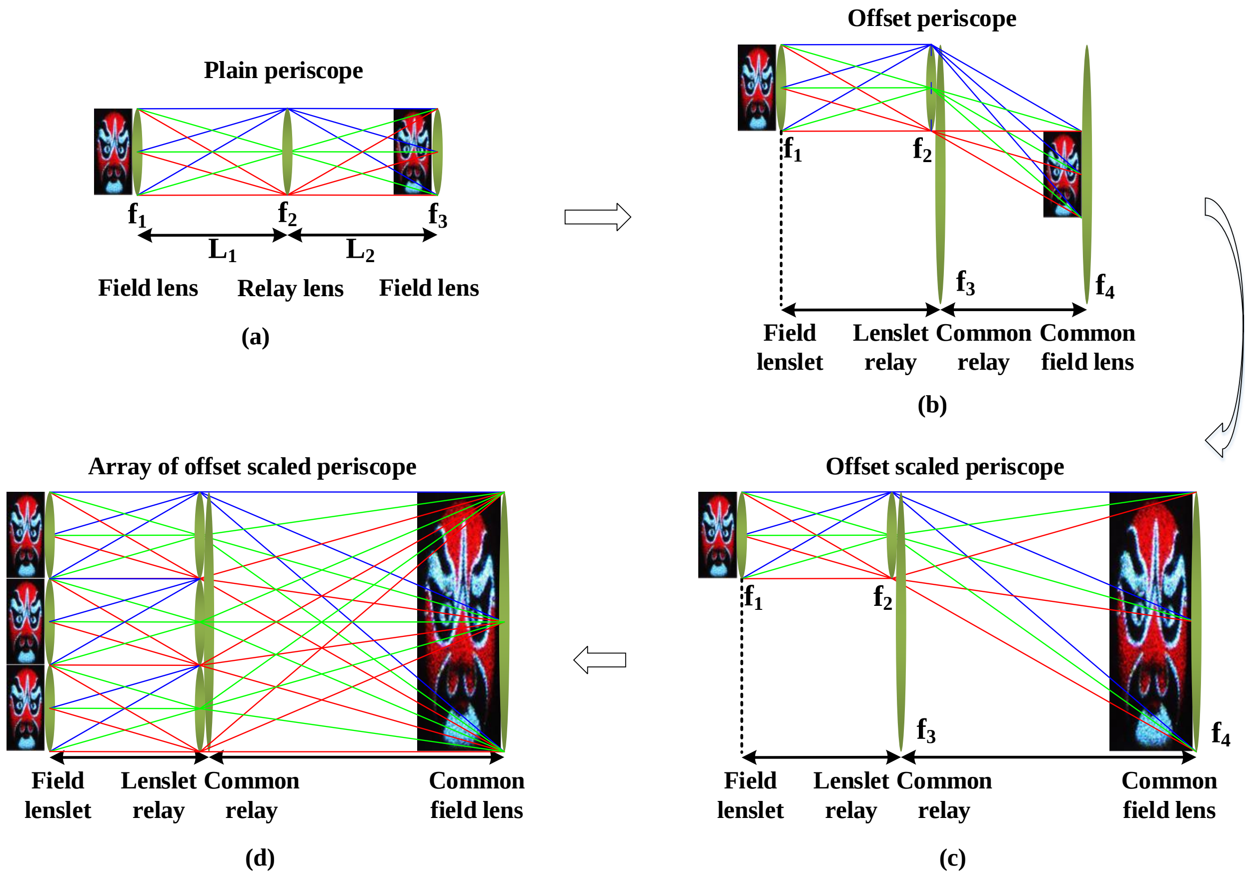

Fig. S2. (a) Plain periscope relay optics, (b) offset periscope, (c) scaled offset periscope, and (d) the array of scaled offset periscope.

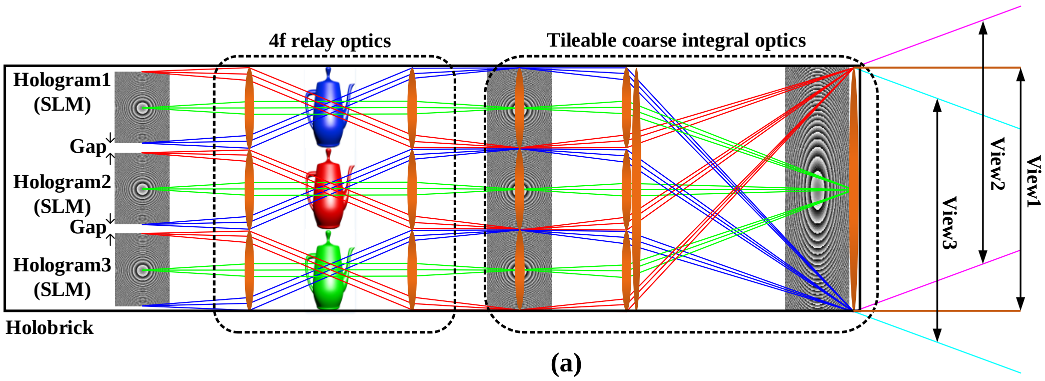

(a)

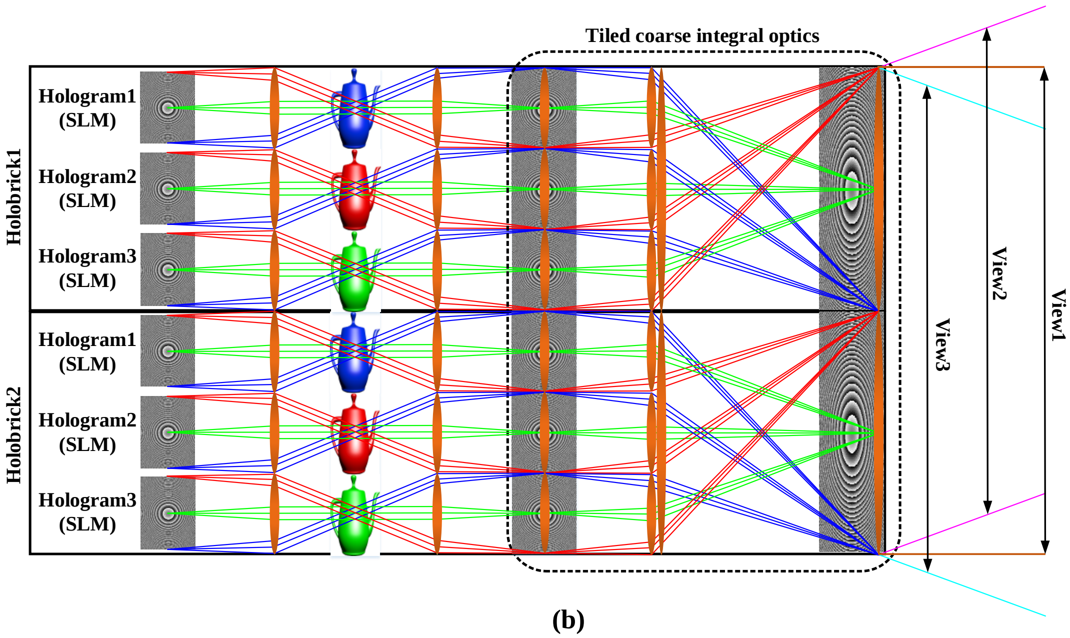

(b)

Fig. S3. All-optical angular and spatial tiling of static holobricks: (a) static holobrick with static tileable CIH, and (b) two static holobricks partially tiled.

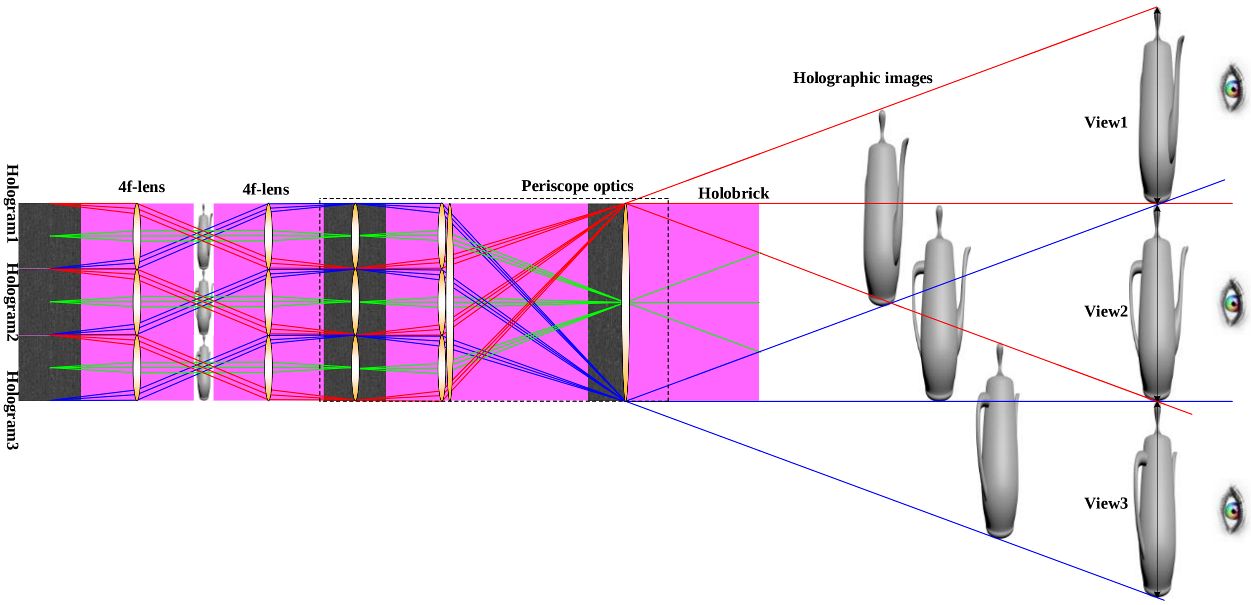

Fig. S4. Displaying images at different viewing angles using a static holobrick with periscope optics.

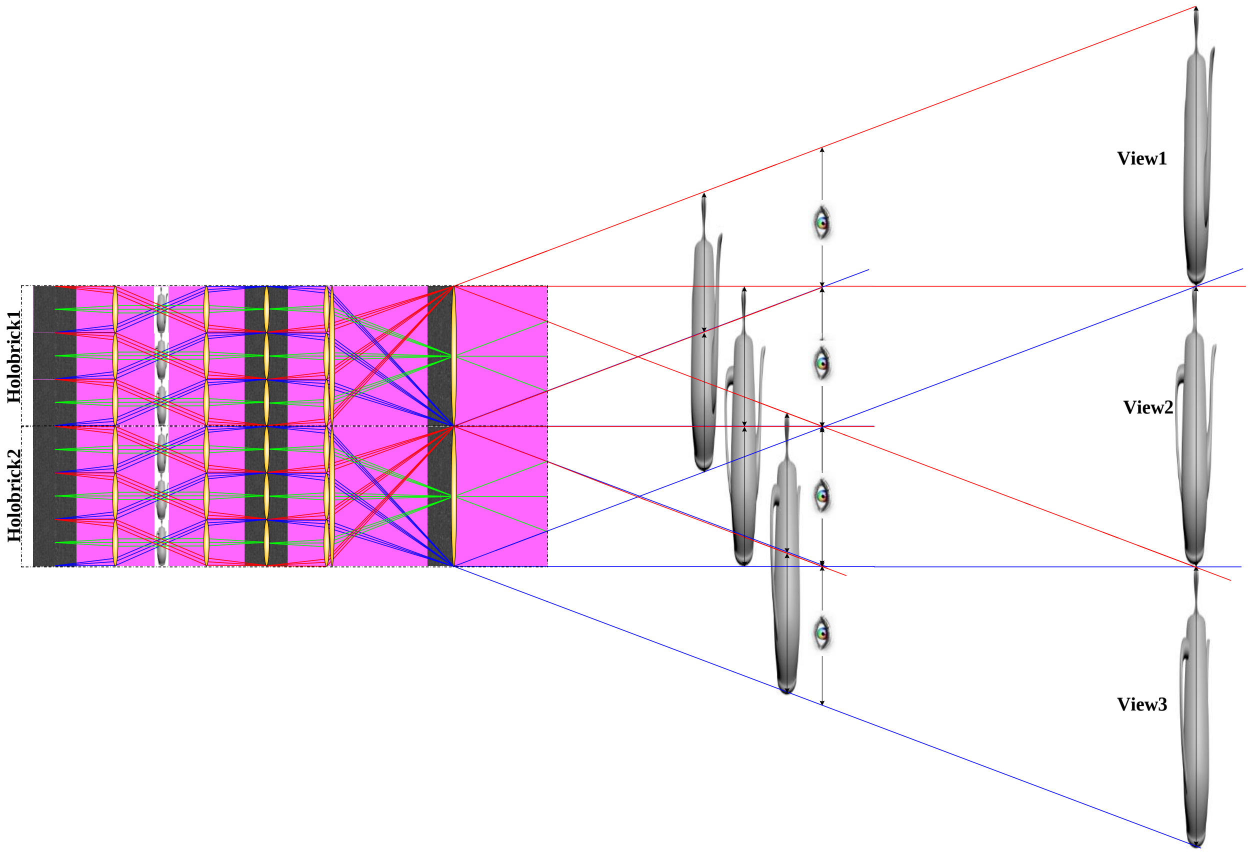

Fig. S5 Two static holobricks to display the double holographic image size with the same the field of view.

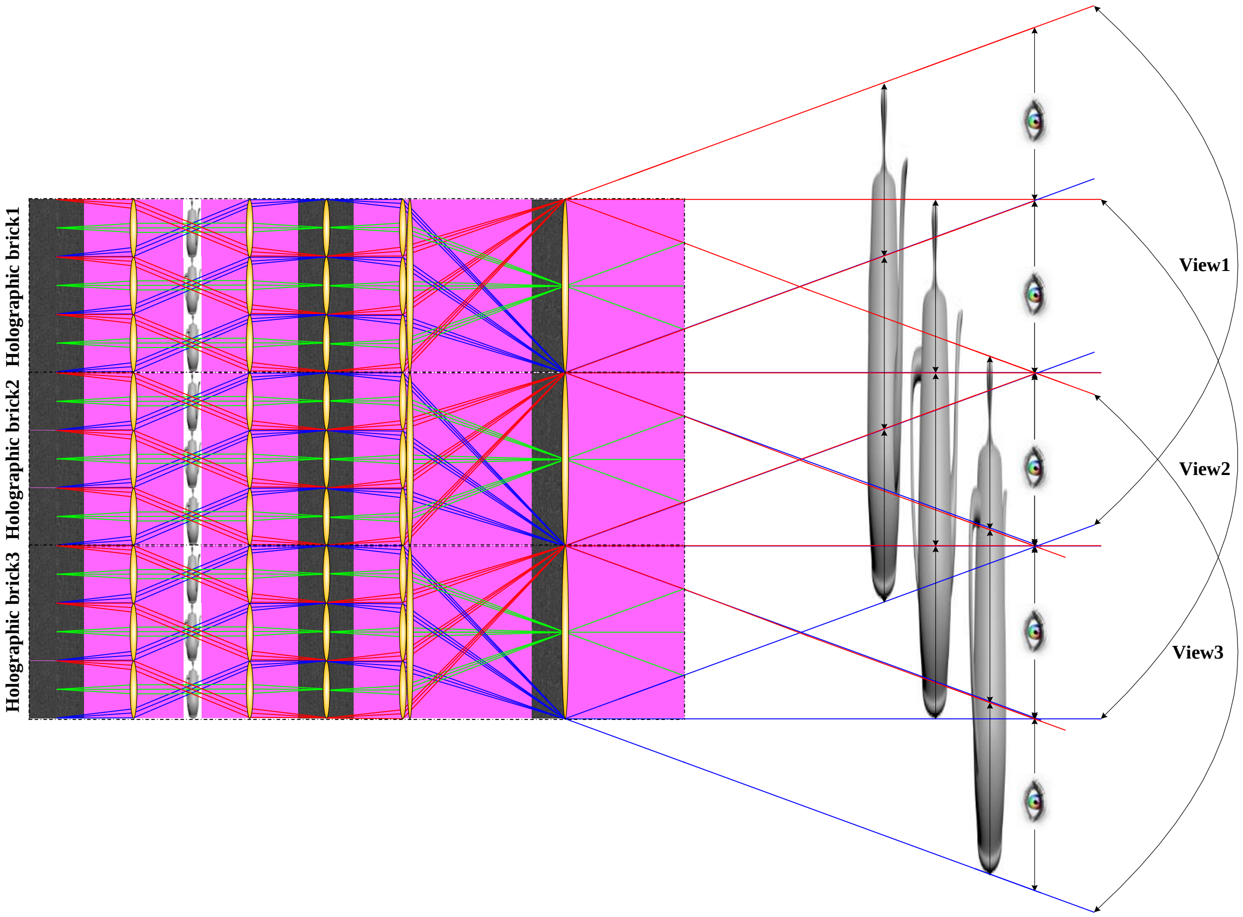

Fig. S6. An example of spatial tiling of three static holobricks with the array of scaled periscope optics to display a large object with the same viewing angle.

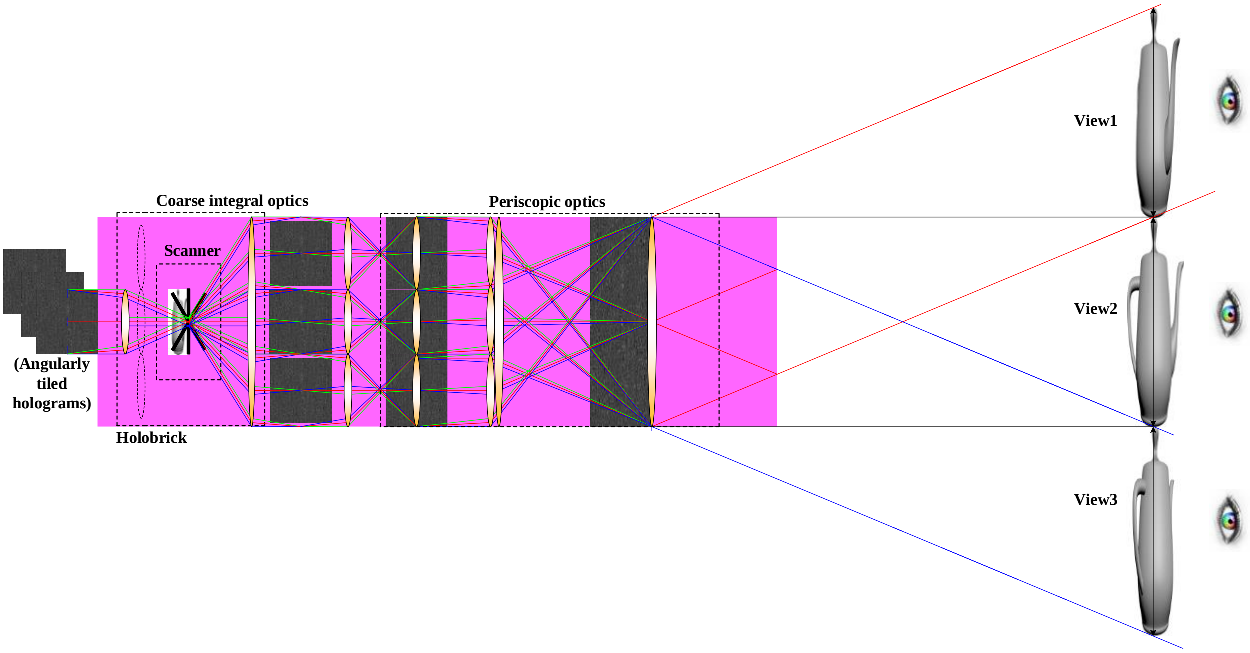

Fig. S7. Displaying images at different viewing angles using a dynamic holobrick with periscope optics.

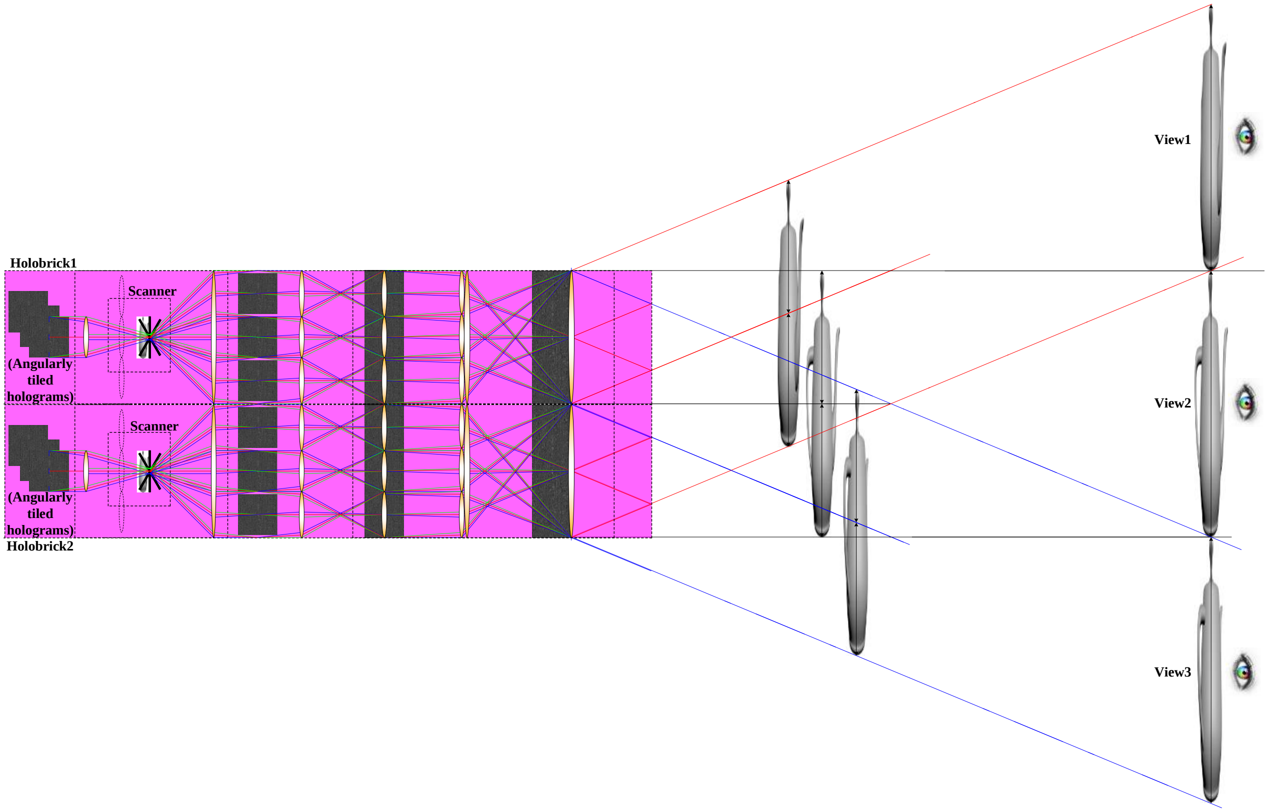

Fig. S8. The all-optical spatial tiling of two dynamic holobricks to display a large object.

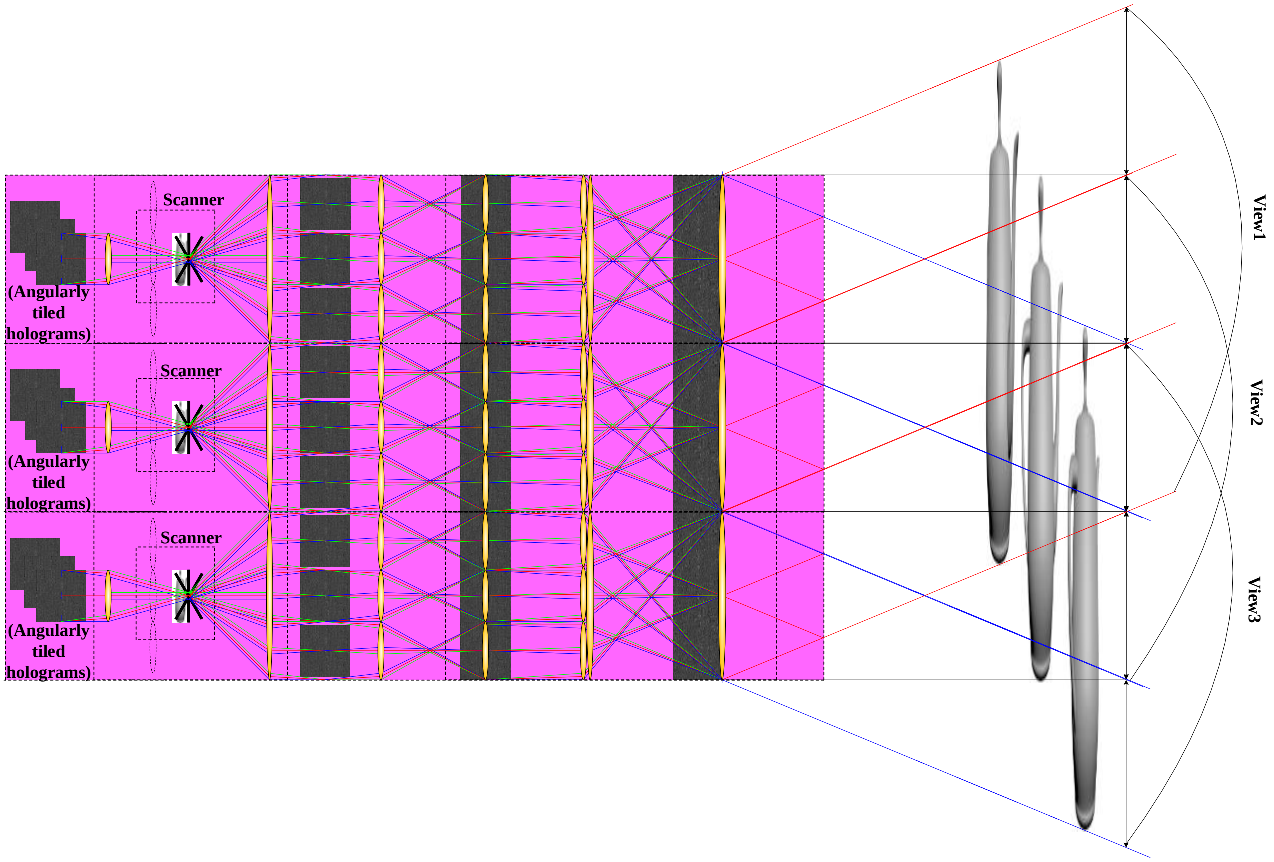

Fig. S9. An example of spatial tiling of three dynamic holobricks to display a large object, which can achieve the same size and viewing-angle results as the three static tiled holobricks.

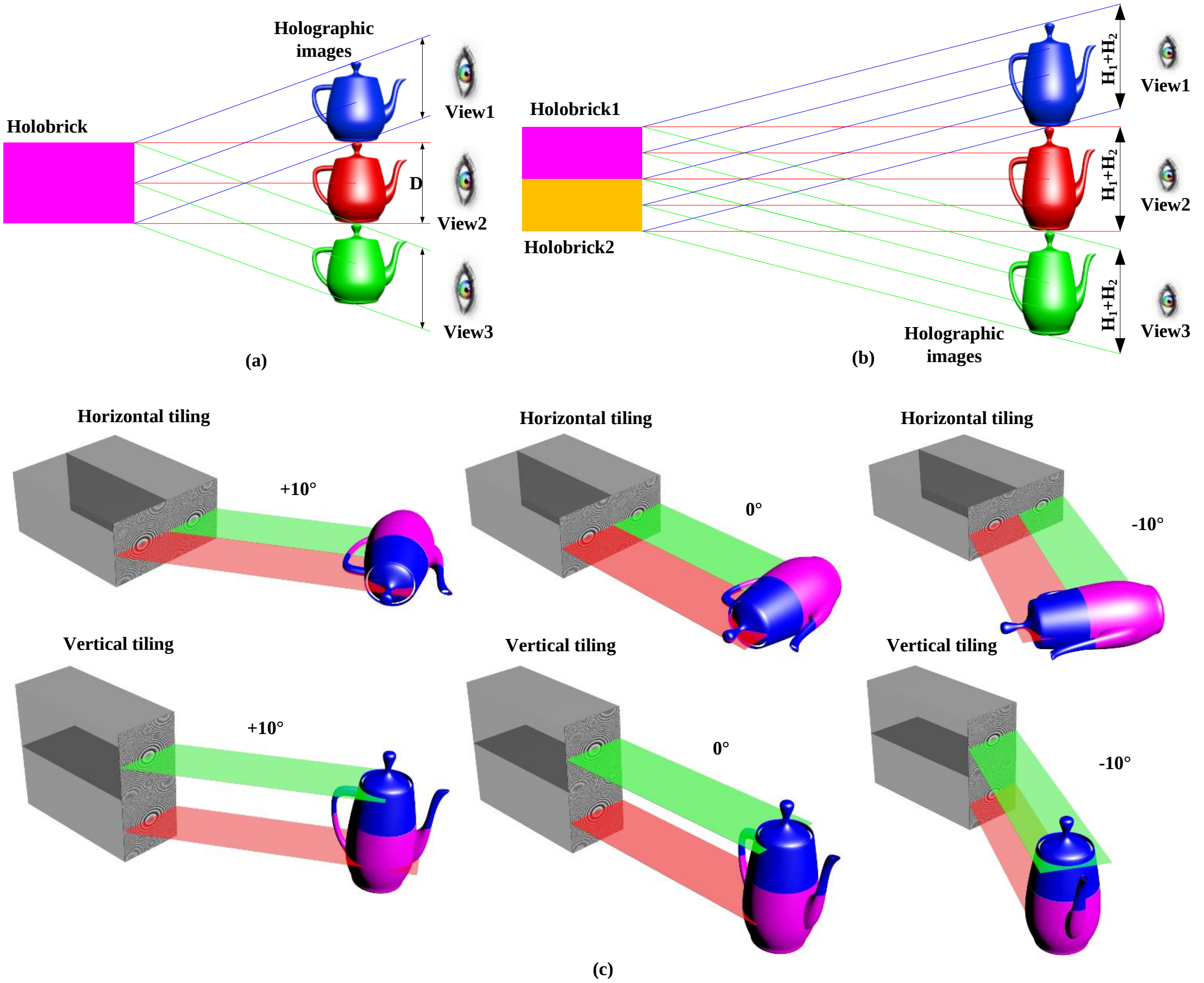

Fig. S10. The allocation examples of the FOV and size of tiled holo-bricks: (a) the FOV and size arrangement of single holobrick, (b) the tiling of FOV and size of two spatially tiled holobricks, and (c) the 3d forms of spatial tiling of an object using two holobricks tiled in the horizontal or vertical direction at different FOVs.

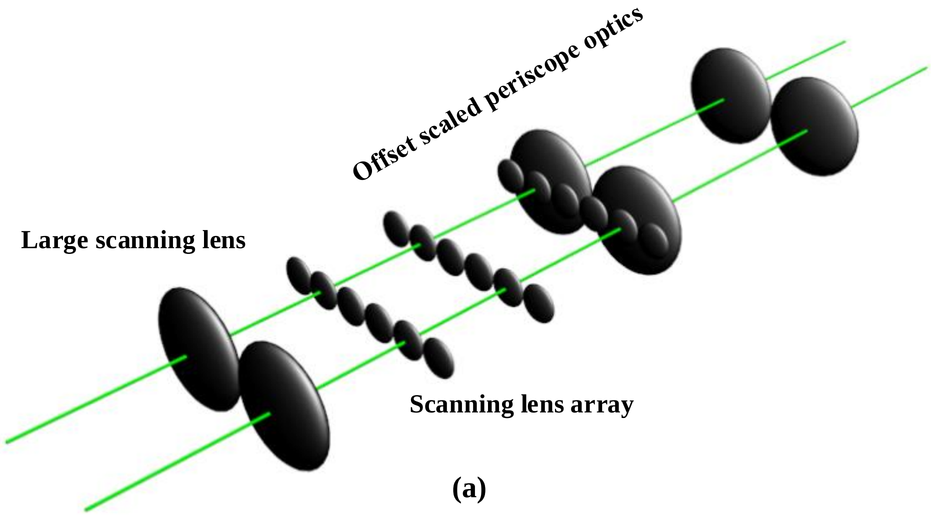

(a)

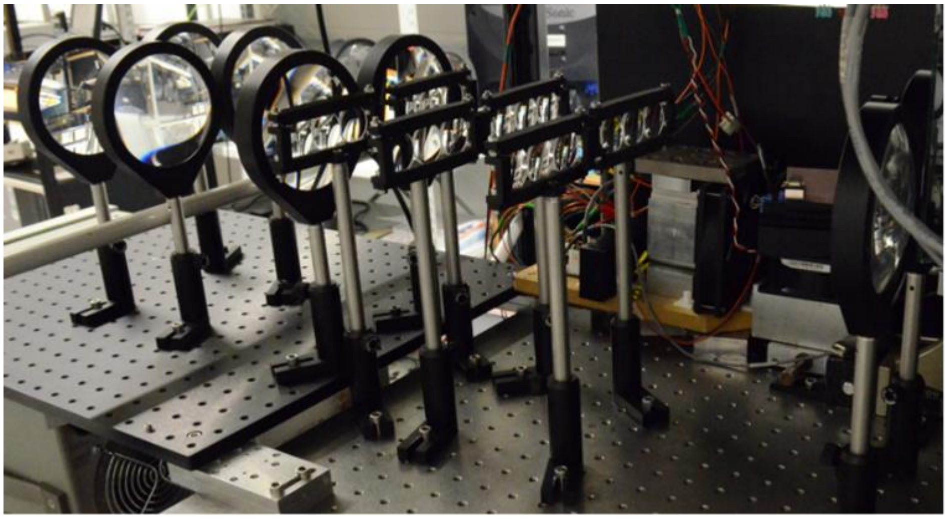

(b)

Fig. S11. The sub-part of the proof-concept-system: (a) offset scaled periscope optics of two holo-bricks, and (b) experimental set up.

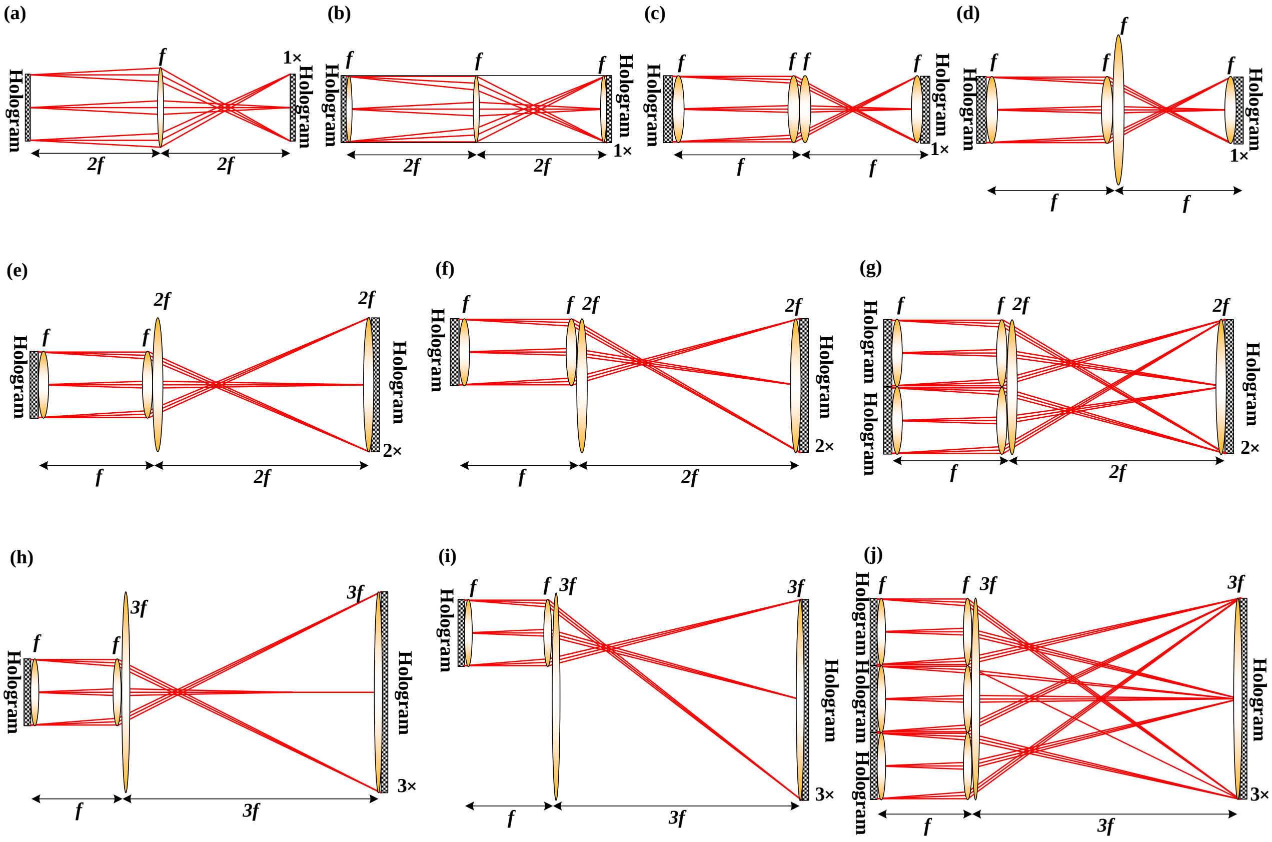

Fig. S12. The fabrication process of an array of offset scaled periscope optics: (a) A standard 2*f*-system can relay a hologram, (b) the plain periscope system with a field lens, which can keep the rays confined to their bundles, (c)-(d) the double lens structure replaces the relay 2*f*-lens in the plain periscope system, (e) the scaled periscope optics using dual lenses achieved the doubling hologram size, (f) the offset periscope optics achieves a doubling hologram size, (g) the array of scaled periscope optical array has two viewing angles, (h) the scaled periscope optics has a triple hologram size, (i) the offset scaled periscope optics obtains a triple hologram size, and (j) the array of the scaled periscope optical array has three viewing angles.

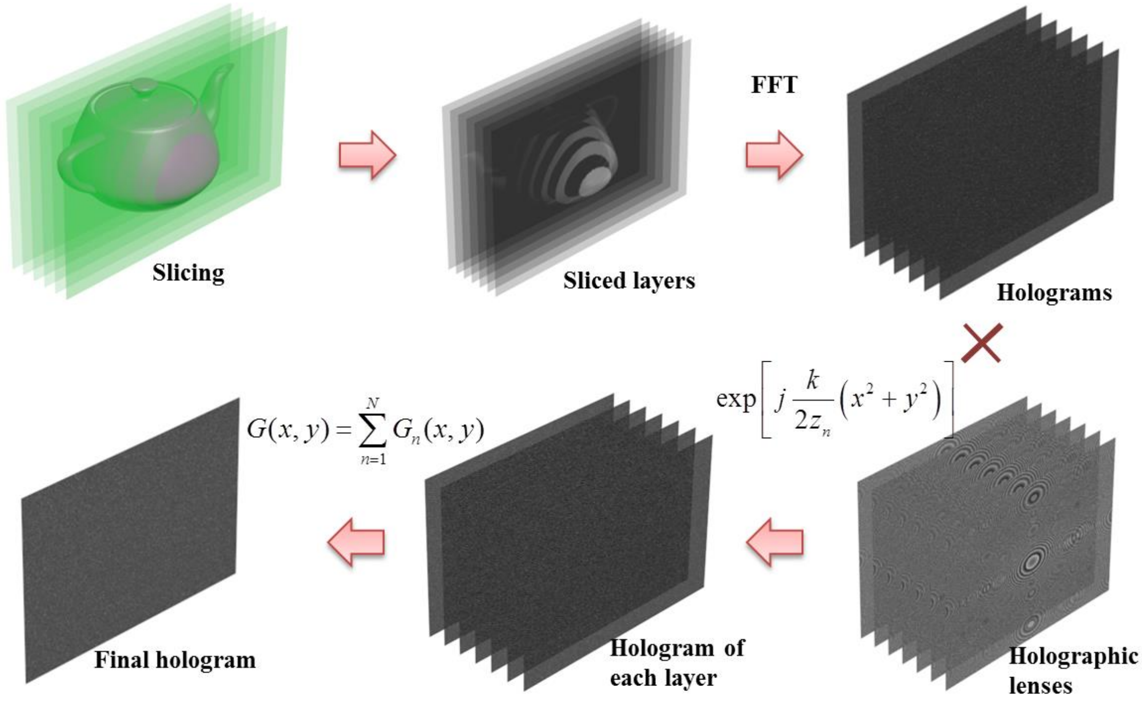

Fig. S13. Hologram generation algorithm for holobricks: each layer is transformed into the frequency domain; a holographic lens is attached behind each layer hologram; the final hologram is the sum of all layer hologram.

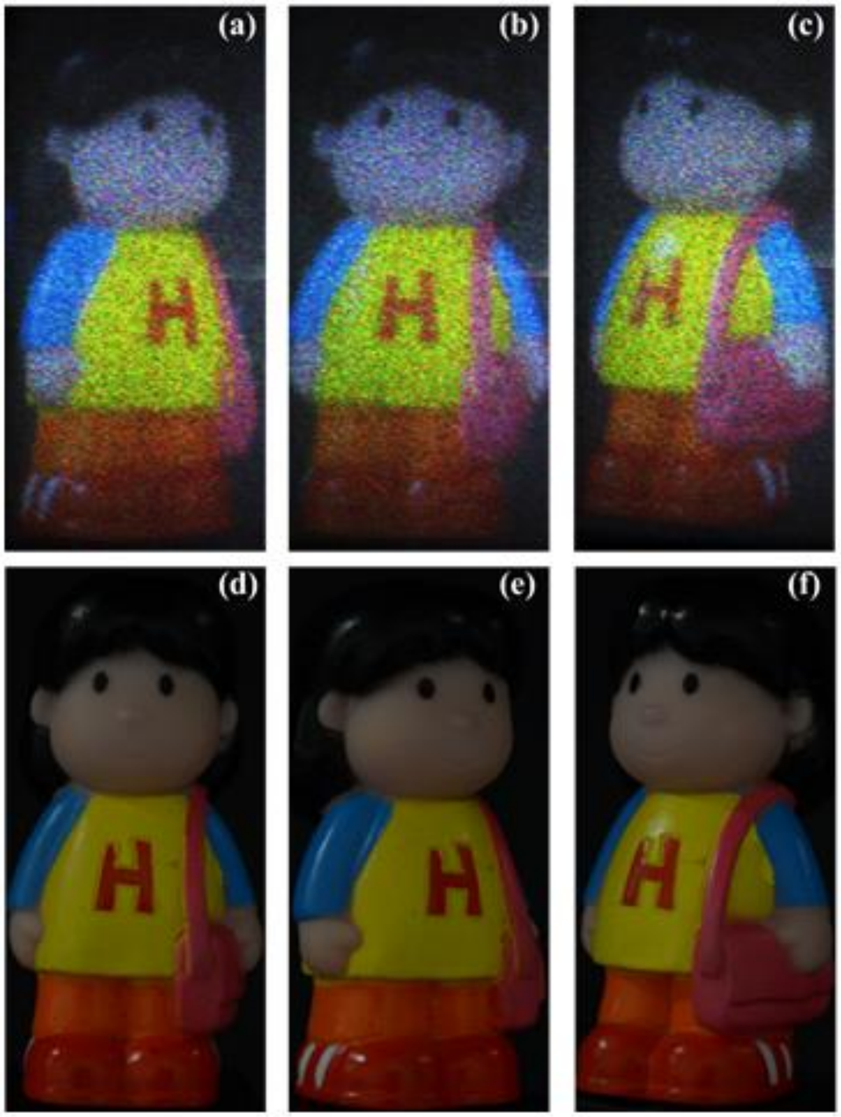

Fig. S14. Reconstructed holographic images of a toy girl with holobricks at the viewing angles of (a)-(c) 0°, +20°, and -20°, with the original toy girl object captured by a camera at the same viewing angles of (d)-(f) 0°, +20°, and -20°, respectively.

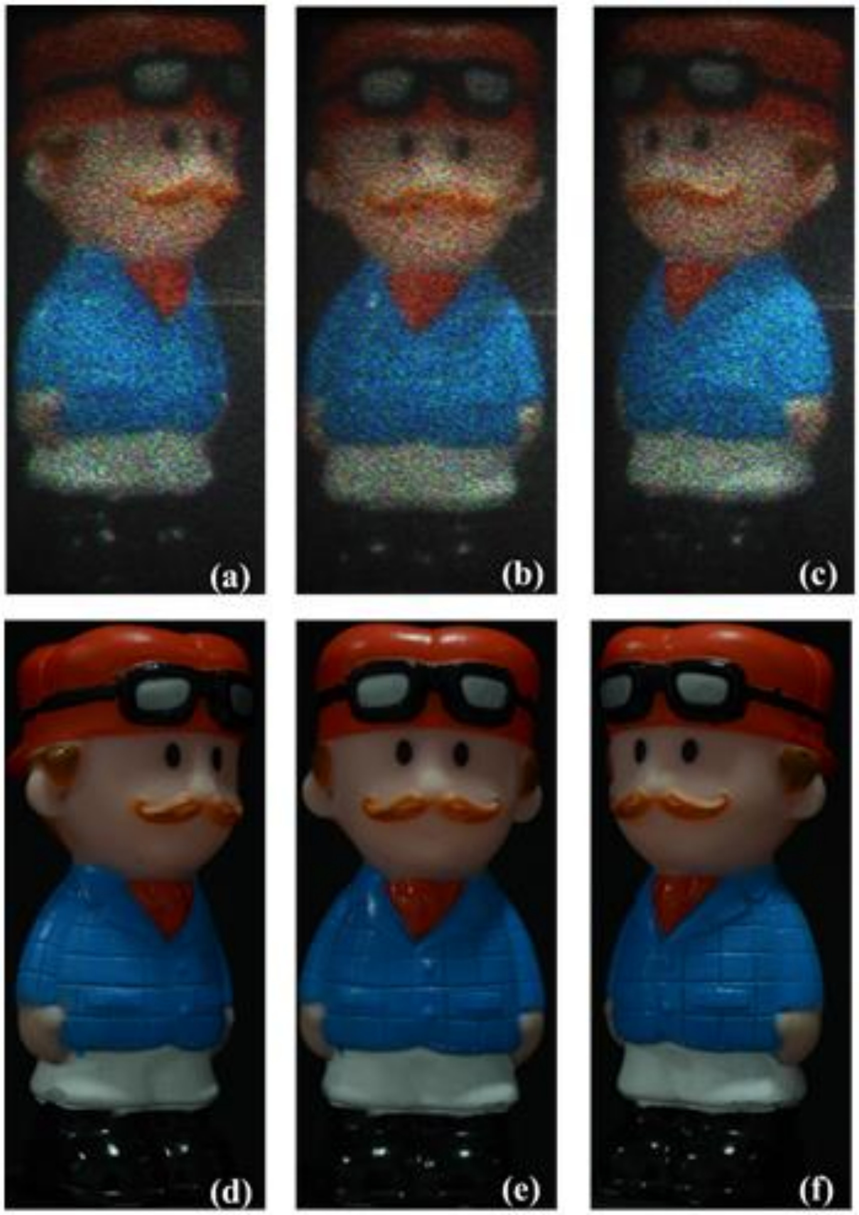

Fig. S15. Reconstructed holographic images of a 3D toy man with holobricks at viewing angles of (a)-(c) 0°, +20°, and -20°, with the original toy man object captured by a camera at the same viewing angles of (d)-(f) 0°, +20°, and -20°, respectively.

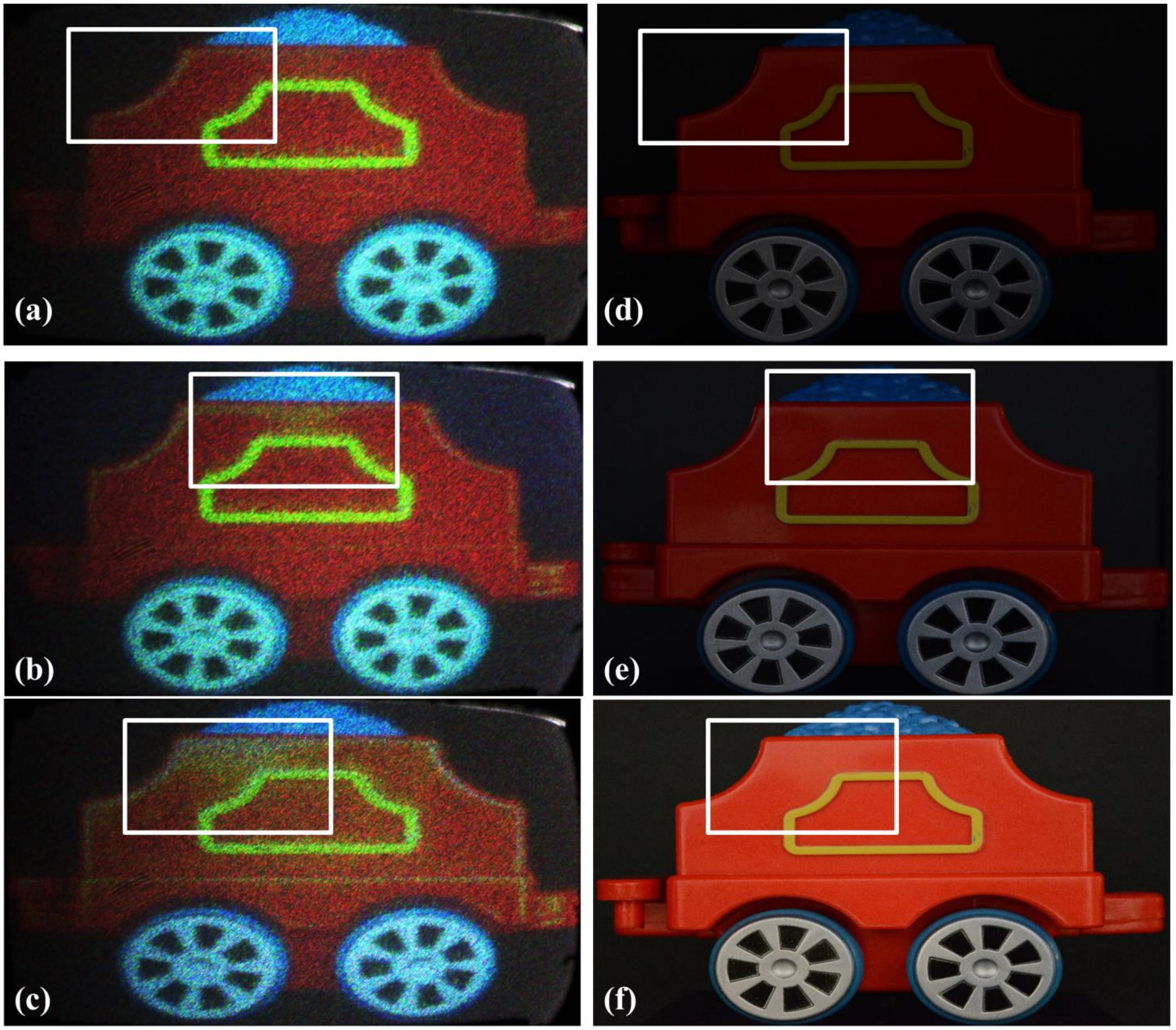

Fig. S16. Reconstructed holographic images (a)-(c) using original images with different sensitivity, where the original images (d)-(f) of a toy train object are captured by a camera under different exposure times. Details of the surface brightness of the toy train can be reconstructed by the holobrick displays, which indicate the holobricks have a high sensitivity performance.

S2. Computer-generated hologram (CGH) generation algorithm for holobricks

In the holobrick display system, the periscope optical array is used to relay the hologram on the DMD plane to the holobrick face. There is not a Fourier lens to reconstruct the holograms of different depths. We use computed holographic lenses added to the Fourier transform of the sliced depth images to generate the different desired-depth holographic images. The holographic patterns on the DMD are relayed and angularly multiplexed by the periscope optical array to the face of the holobrick. The relayed hologram from the periscope optical array can directly reconstruct the holographic images. The hologram generation algorithm is composed of four steps. First, a 3D object image at a viewing-angle position is sliced into multiple depth layers based on its depth map. Here, we use $G=[G_1,G_2,\ldots,G_n,\ldots,G_N]$ to express the depth layers, where N is the total depth number. Second, each depth image is attached by a random phase. Third, the corresponding Fourier holographic pattern of each depth image is generated by a Fourier transform operation. Here, we use P_n to express the Fourier holographic pattern, P_n=FFT(G_n). Fourth, each depth hologram is attached by a computed holographic lens as:

$$Q_n(x,y) = P_n \times \exp\left[j\frac{k}{2}\left(\frac{x^2}{z_{nx}} + \frac{y^2}{z_{ny}}\right) \right] \quad \text{(S1)}$$

This method is a layer algorithm when the depth information is considered. The “attached” holographic lens is computed as part of the hologram, and scanned with the SLM into the array, removing the need for a coarse integral optics’ lenslet array as well. The generation algorithm is shown in Supplementary Fig. S13.

S3. The occlusion or accommodation for the holobrick system

Same as the other holographic displays, holograms can produce the accommodation depth cues and occlusions. These two features have a direct relationship with the hologram generation algorithm. A holographic display system can provide the capability of these two effects. With the same capability as our previous scalable full-bandwidth dCIH systems, our graphics rendering approach for the presented holobrick system allows the incorporation of all appropriate depth cues and occlusion in the generated holograms for angular tiling. The presented holobrick system can display holographic images with accommodation cues. This accommodation cue capability is the same as our previous scalable full-bandwidth dCIH systems. A significant feature of the CIH systems is the trade-off between the resolution of accommodation cue and hologram generation calculation speed. Under the acceptable calculation consumption, the accommodation cue resolution is determined by the object and the human visual system. For example, a total depth of an object is 10 cm. When the layer number is 10~20, the depth resolution is 5 mm~10 mm. The depth resolution can satisfy the eye accommodation and the system can provide smooth accommodation cues.

In our holobrick system, the nature of angularly tiled CIH holograms can be fully utilized to produce view-dependent holograms. Each sub-hologram generated by our layer-based algorithm can project a voxel emitting light isotropically within its small FOV zone. The tileable coarse integral optics can provide angularly tiled view zones independent of each other. The independence of the view zones allows each sub-hologram to be computed separately, independently of other sub-holograms for efficient and parallel computation. This allows us to render and display occlusion /disocclusion effects, to view dependent shading and lighting, as well

as to prevent layering artifacts with off-axis viewing of layered holograms. Our computer-generated imagery color and depth renderings of the 3D model from different viewpoints can provide view-dependent lighting, occlusion/disocclusion handling, and layer slicing.

S4. Objective evaluations of the holobrick display system

We also objectively evaluate display system performance using the following several measurements. Firstly, the reconstructed holographic image quality from the holobrick system is measured by eight parameters: contrast, edge intensity (EI), average gradient (AG), entropy, variance, luminosity, homogeneity, and mean structural similarity index (MSSI). Table S1 shows the measurement results at different FOV positions. From the measure results, the reconstructed images can obtain high performance in terms of contrast, EI, AG, entropy, variance, luminosity, homogeneity, and MSSIM. For example, the reconstructed and original images have a similar performance in the contrast. These measurement results indicated that the proposed system can achieve a high display performance.

Table S1 the measurement results of the reconstructed image quality of the display systems

**Parameters**	**Reconstructed image**			**Original image**		
	-20°	0°	+20°	-20°	0°	+20°
**Contrast**	230.2500	233.7500	229.7500	230.7500	237.5000	234.0000
**EI**	38.8893	40.3752	39.7868	17.6858	18.4603	18.2444
**AG**	3.9690	4.0557	3.8703	1.6334	1.7049	1.6833
**Entropy**	6.9444	7.0049	6.9651	5.1246	5.1326	5.1698
**Variance**	46.7497	46.1531	44.9346	42.8775	43.9718	43.0426
**Luminosity**	56.6442	58.2692	58.2195	33.7100	34.9410	34.3365
**Homogeneity**	0.8921	0.8860	0.8873	0.9628	0.9650	0.9625
**MSSIM**	N/C	N/C	N/C	0.3009	0.3073	0.3065

We also measure a (modulation transfer function) MTF value of the holobrick system because the MTF can describe the complex response of an optical system. Currently, multiple MTF measurement methods have been developed. Different targets have been reported for estimating the MTF of optical imaging systems. A slanted-edge method specified in the standard ISO 12233 [6]-[8] is widely used for the MTF evaluation of an optical system. However, this method is suitable for general optical systems and not for a holographic display system. Here, we utilize speckles from the holographic images as point sources to measure the MTF. Fig. S17 shows the measured MTF curve. From the measurement results, the display system exhibited a good MTF performance. At the normalized frequency of 0.5, the MTF can achieve 0.44. This value represents the high optical performance of the holographic system.

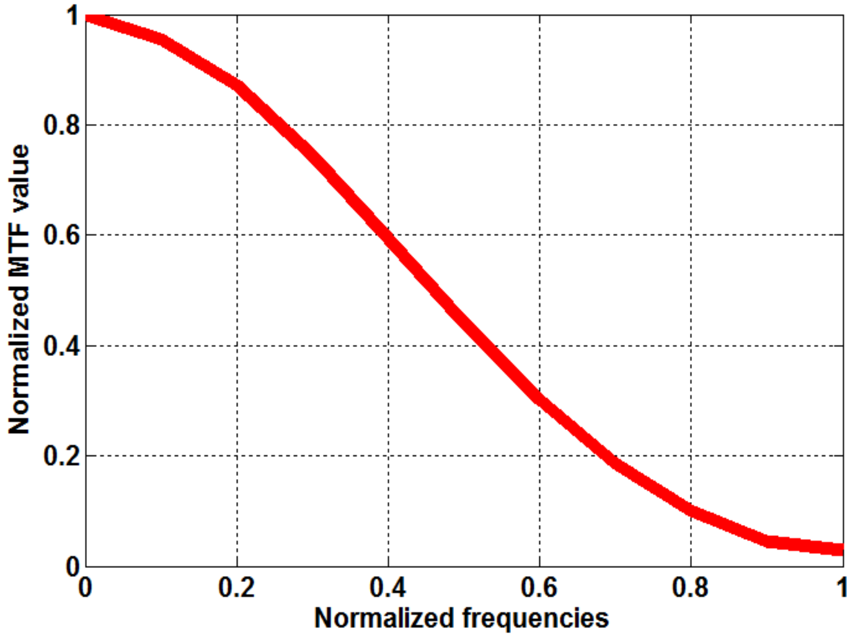

Fig. S17. The MTF estimation results for the holobrick system

Finally, we also analyze other performances regarding the optical efficiency, the smallest viewing-angle matching, and color size matching for the holobrick system. A holobrick system

needs a high-bandwidth physical carrier of holographic fringe patterns. The hologram carrier can be implemented by different spatial light modulators, such as phase-only LOCS, deformable mirrors (DM), and digital micro-mirror devices (DMD). Our experimental holobrick system utilized a high-bandwidth DMD (Discovery™ 4100 chip) as the hologram carrier. From the objective measurements, the optical efficiency of the DMD can achieve 89% in the visible range. In an experimental holobrick system, the DMD has an XGA resolution (1,024 × 768) with a pixel pitch size of 13.7 μm. A hologram presented on the SLM, with at least two pixels per finest fringe period, have a diffraction range of 1.38° × 1.38°, 1.11° × 1.11°, 0.94° × 0.94° about the center of the field of view for 660 nm, 532 nm, and 450 nm laser light sources, respectively. When a viewer observes holographic images outer of these diffraction angles, the content mismatch can be perceived. In a holobrick, the optical scanning angle of ± 20° can tile 50 sub-holograms per row horizontally. Each sub-view for angular tiling is ±0.4° that less than the diffractive angle of the DMD. Therefore, there is no situation of the content mismatch. Accordingly, the sub-holograms are scanned into six vertical rows and six vertical sub-holograms (± 0.53° each) are obtained. In the future, a high-speed phase-only LOCS is a potential hologram carrier for the optimization of holobricks. In addition, in our holobrick system, each holographic frame is composed of three colors. The color size matching is an important aspect because different wavelengths cause different image sizes (scaling). Based on the diffraction imaging theory, the horizontal and vertical interval of hologram can be expressed by $\Delta x=\lambda d/m\Delta a$, $\Delta y=\lambda d/n\Delta b$, where λ represents the wavelength, d denotes the reconstructed hologram distance, m and n denotes the pixel number of DMD, and Δa and Δb represents the pixel size of DMD. The color size of three holographic images has a ratio relationship as: X_R=1.24*X_G, X_R=1.46*X_B. This ratio relationship is inserted

into our hologram calculation.

S5. A video record example of holographic displays with different FOV angles for a holobrick system

We demonstrated a video record example of holographic displays with different parallax images for the holobrick system. This example also showed a complete holographic display flow from the hologram generation and holographic displays for a holobrick system. Figure S18 shows the complete record process of the holographic display with different viewing angles (i.e., parallax) using a camera. First, we use 3ds Max to design a 3D object model to produce different parallax images. The 3D object model is a curved surface composed of 16 sub-surfaces. Each sub-surface is designed with a capital letter. Each sub-surface has a small viewing angle of 2.5°. Thus, the total FOV angle of the designed 3D object is 40°. Second, each angular-view hologram is calculated from its image Fourier transformation using the hologram generation algorithm. Third, all sub-holograms are presented on the SLMs of the tiled holobrick system. The sub-scanning system of the holobrick can scan low SBP sub-holograms to form the hologram array for the integral optics. Fourth, we placed a camera in front of the holobrick system to record the holographic image display process at the different FOV positions. In the recording process, the camera is moved along the perpendicular direction of the optical axis from the left FOV to the right FOV. The holographic images of different viewing angles are recorded. A recorded video is shown in Supplementary video materials. From the video, we can observe the different parallax holographic images at different times.

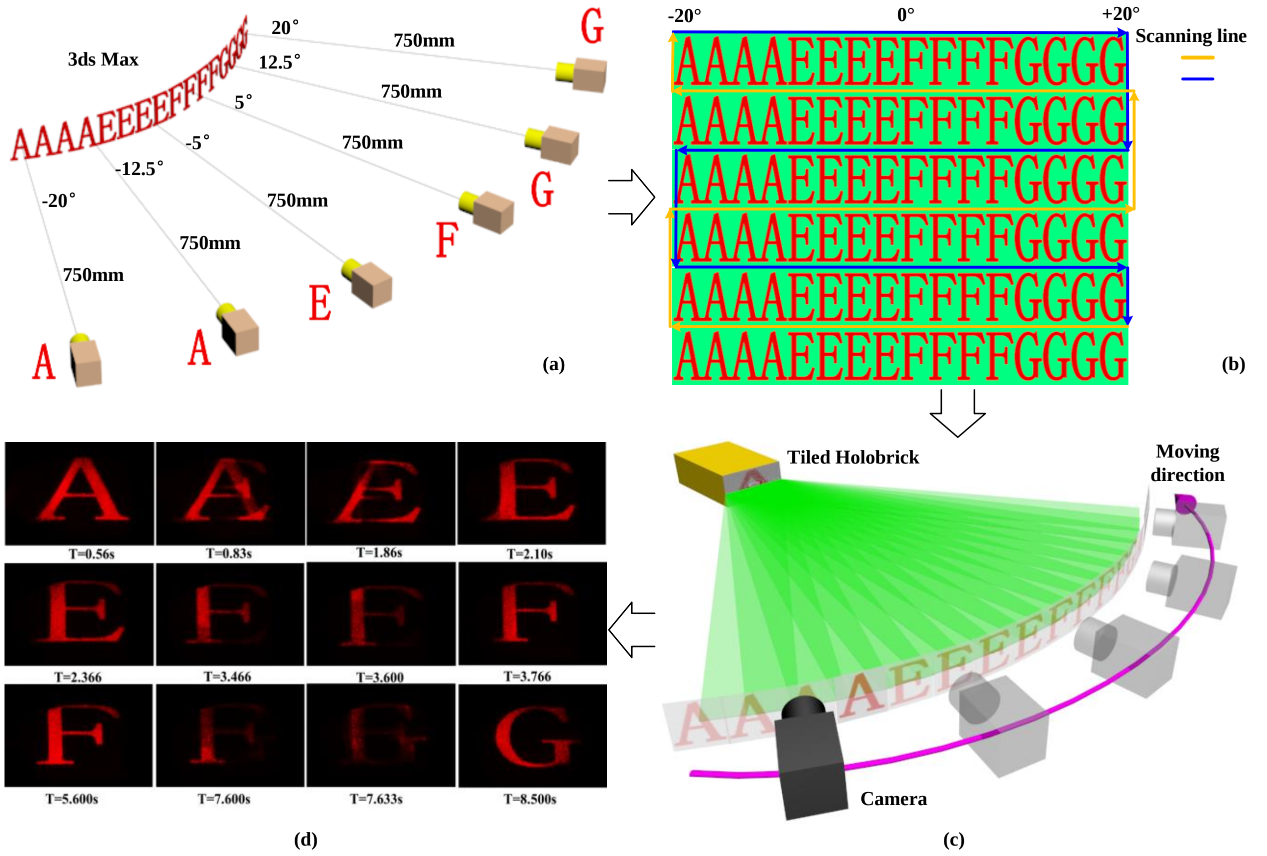

Fig. S18. An example of a holographic image display for a tiled holobrick system, where a camera was used to record the display process at the different FOV positions: (a) a 3D model curved surface designed by 24 sub-surfaces (capital letters: A, E, F, and G) under the 3ds Max platform; (b) The scanning subsystem of the holobrick system can scan low SBP sub-holograms to form the hologram array for the tileable integral optics; (c) In the front of the holobrick, a camera is moved from the left FOV to the right FOV to record the holographic images; (d) Different parallax holographic images are observed at the different times.

References

[1] Chen, J. S., Smithwick, Q. Y. & Chu, D. P. Coarse integral holography approach for real 3D color video displays. *Optics Express* **24**, 6705-6718 (2016).

[2] Chen, J. S. *et al*. Auxiliary Resonant Scanner to Increase the Scanning Capability for Coarse Integral Holographic Displays. *Chinese Optics Letters* **15**, 040901 (2017).

[3] Li, J., Smithwick, Q. Y. & Chu, D. P. Full bandwidth dynamic coarse integral holographic displays with large field of view using a large resonant scanner and a galvanometer scanner. *Optics Express* **26**, 17459-17476 (2018).

[4] Li, J., Smithwick, Q. Y. & Chu, D. P. Bandwidth utilization improvement methods of Coarse Integral Holographic video displays. Proceedings of Imaging and Applied Optics 2018, Digital Holography and Three-Dimensional Imaging, Orlando: Optical Society of America, 2018, DTh3D-6 .

[5] Li, J., Smithwick, Q. Y. & Chu, D. P. Scalable coarse integral holographic video display with integrated spatial image tiling. *Optics Express* **28**, 9899-9912(2020).

[6] Estribeau, M. & Magnan, P. Fast MTF measurement of CMOS imagers using ISO 12333 slanted-edge methodology. Proceedings of SPIE 5251, Detectors and Associated Signal Processing, France: SPIE, 2003, 5251.

[7] Photography - electronic still picture imaging - resolution and spatial frequency responses, Document ISO 12233:2017, 2017.

[8] Hwang, H. *et al*. MTF assessment of high resolution satellite images using ISO 12233 slanted-edge method. Proceedings of SPIE 7109, Image and Signal Processing for Remote Sensing XIV, Wales: SPIE, 2008, 710905.
